# Exercise Protects Against Defective Insulin Signaling and Insulin Resistance of Glucose Transport in Skeletal Muscle of Angiotensin II-Infused Rat

**DOI:** 10.3389/fphys.2018.00358

**Published:** 2018-04-11

**Authors:** Juthamard Surapongchai, Yupaporn Rattanavichit, Jariya Buniam, Vitoon Saengsirisuwan

**Affiliations:** Exercise Physiology Laboratory, Department of Physiology, Faculty of Science, Mahidol University, Bangkok, Thailand

**Keywords:** angiotensin II, insulin signaling, insulin resistance, MAPK, soleus muscle, voluntary exercise

## Abstract

**Objectives:** The present study investigated the impact of voluntary exercise on insulin-stimulated glucose transport and the protein expression and phosphorylation status of the signaling molecules known to be involved in the glucose transport process in the soleus muscle as well as other cardiometabolic risks in a rat model with insulin resistance syndrome induced by chronic angiotensin II (ANGII) infusion.

**Materials and Methods:** Male Sprague-Dawley rats were assigned to sedentary or voluntary wheel running (VWR) groups. Following a 6-week period, rats in each group were subdivided and subcutaneously administered either normal saline or ANGII at 100 ng/kg/min for 14 days. Blood pressure, glucose tolerance, insulin-stimulated glucose transport and signaling proteins, including insulin receptor (IR), insulin receptor substrate 1 (IRS-1), Akt, Akt substrate of 160 kDa (AS160), AMPKα, c-Jun NH_2_-terminal kinase (JNK), p38 MAPK, angiotensin converting enzyme (ACE), ANGII type 1 receptor (AT1R), ACE2, Mas receptor (MasR) and oxidative stress marker in the soleus muscle, were evaluated.

**Results:** Exercise protected against the insulin resistance of glucose transport and defective insulin signaling molecules in the soleus muscle; this effect was associated with a significant increase in AMPK Thr^172^ (43%) and decreases in oxidative stress marker (31%) and insulin-induced p38 MAPK Thr^180^/Tyr^182^ (45%) and SAPK/JNK Thr^183^/Tyr^185^ (25%), without significant changes in expression of AT1R, AT2R, ACE, ACE2, and MasR when compared to the sedentary rats given ANGII infusion. At the systemic level, VWR significantly decreased body weight, fat weight, and systolic blood pressure as well as improved serum lipid profiles.

**Conclusion:** Voluntary exercise can alleviate insulin resistance of glucose transport and impaired insulin signaling molecules in the soleus muscle and improve whole-body insulin sensitivity in rats chronically administered with ANGII.

## Introduction

Insulin resistance of skeletal muscle is the reduced ability of insulin to stimulate glucose uptake into skeletal muscle, the major site of glucose disposal. Skeletal muscle insulin resistance frequently occurs in parallel with other cardiometabolic risks including glucose intolerance, hyperinsulinemia, dyslipidemia, essential hypertension, and central obesity. Together, these multifaceted conditions are known as insulin resistance syndrome, a major risk factor of diabetes and cardiovascular diseases (Reaven, 2005; DeFronzo and Tripathy, 2009). Angiotensin II (ANGII), a component of the renin-angiotensin system (RAS), has been demonstrated as a cause of insulin resistance (Carlsson et al., 1998; Luther and Brown, 2011). An excess of ANGII induces insulin resistance and impairs glucose homeostasis through the classical RAS pathway by modulating the angiotensin-converting enzyme/ANGII/angiotensin II type I receptor (ACE/ANGII/AT1R) axis (de Kloet et al., 2010). Clinical observation has demonstrated that the plasma ANGII levels in patients with essential hypertension are higher when compared to healthy individuals (Catt et al., 1969). In addition, it has been reported that a higher level of plasma ANGII is associated with insulin resistance and diabetic severity (Nicola et al., 2001). Interestingly, the adverse effects of ANGII could be counteracted when the novel RAS pathway was enhanced via proteins in the ACE2/angiotensin-(1-7)/Mas receptor (MasR) axis (Santos et al., 2008; Prasannarong et al., 2012; Santos and Andrade, 2014). For instance, activation of the ACE2/ANG-(1-7)/MasR axis led to an improvement in glucose and lipid metabolism (Ferrario et al., 2005; Santos et al., 2008, 2013; Prasannarong et al., 2012; Echeverria-Rodriguez et al., 2014; Santos and Andrade, 2014).

Exercise is a non-pharmacological intervention for various pathological conditions (Golbidi et al., 2012; Pattyn et al., 2013; Karjalainen et al., 2015). In insulin-resistant condition, regular exercise training improves skeletal muscle insulin sensitivity by modulating a variety of molecular signaling factors including calcium, nitric oxide, glycogen, hypoxia, AMPK, AICAR, mitogen-activated protein kinases (MAPKs) and oxidative stress (Richter and Hargreaves, 2013; Messina et al., 2015). Exercise training in small animal models can be conducted by using a variety of exercise modalities. In chronic heart failure (Gomes-Santos et al., 2014) and diet-induced obese rats (Frantz et al., 2017), endurance exercise training on a treadmill shifted the balance of RAS toward the novel pathway in skeletal muscle. While the favorable effect of exercise training has been shown mostly in obese animals (Cortez et al., 1991; Jungersten et al., 1997), the potential benefits of exercise training in disease models induced by humoral factors have received far less attention. In contrast to forced exercise training, voluntary running on wheels is a less stressful exercise condition since the animals voluntarily run on wheels with no aversive stimuli; however, the impact of voluntary exercise on cardiometabolic risks and the precise mechanisms are less consistent possibly due to a variation in running distances, loads, animal species and pathological conditions (Dumke et al., 2001; Kinnick et al., 2002; Lemieux et al., 2005; Wei Sun et al., 2008; Garvey et al., 2015).

It has been demonstrated that chronic administration of ANGII to rats led to hypertension and insulin resistance of skeletal muscle glucose uptake with multiple post-receptor defects (Lastra et al., 2013; Surapongchai et al., 2017). Nevertheless, it remains to be elucidated how exercise training can modulate the cellular mechanisms underlying ANGII-induced skeletal muscle insulin resistance. In this context, the purpose of this study was to investigate the effects of exercise training by wheel running on insulin-stimulated glucose transport activity and the potential cellular mechanisms in skeletal muscle of ANGII-infused rats. To address this issue, the protein expression and functionality of the signaling molecules known to regulate glucose transport, such as proteins in the insulin signaling, MAPKs and RAS pathways as well as oxidative stress marker, were examined in the soleus muscle of ANGII-infused rats with or without access to running wheel. Moreover, other key features of insulin resistance syndrome, including arterial blood pressure, glucose tolerance, abdominal fat accumulation and lipid profiles, were evaluated.

## Materials and methods

### Animal treatment and voluntary wheel running protocol

This study was carried out in accordance with the recommendations of the International Guiding Principles for Biomedical Research Involving Animals of the Council for International Organizations of Medical Sciences, the Animal Care and Use Committee, Faculty of Science, Mahidol University. The protocol (No. MUSC54-001-245) was approved by the Animal Care and Use Committee, Faculty of Science, Mahidol University.

Eight-week-old male Sprague-Dawley rats, weighing between 260 and 290 g, were supplied by the National Laboratory Animal Center (Nakhon Pathom, Thailand). Rats were housed in a temperature-controlled room at 22°C with a 12/12-h light/dark cycle (light on from 06:00 AM to 06:00 PM) and were allowed to access water and pellet rat chow (Perfect Companion, Samut Prakan, Thailand) *ad libitum*. Rats were randomly assigned into either sedentary (SED) or voluntary wheel running (VWR) groups. Rats were housed in an activity wheel with living chamber (model no. 80859; Lafayette Instruments, Lafayette, IN), and only rats in the VWR groups had free access to activity wheels for 24 h. The wheel revolutions of a single rat activity were monitored by an optical counter sensor attached outside of the running wheel. Data on physical activity including interval counts, maximum and average running speed, and total distance were collected by computer interface and the activity wheel monitor software.

After a 6-week period of sedentary or VWR, rats in each group were subdivided and subcutaneously administered either normal saline (SHAM) or ANGII at 100 ng/kg/min (ANGII) for 14 days. A surgical procedure for subcutaneous implantation of an Alzet osmotic minipumps (model 2002) (DURECT Corporation, Cupertino, CA) was performed under anesthesia following the company's recommended procedure. After 24 h of implantation, rats in the exercise groups retained their free access to running wheels. Body weight, food and water intake were monitored regularly.

### Blood pressure determination

One week before the experiment started, rats were acclimated to the tail-cuff plethysmography apparatus (CODA^TM^ Monitor System, Kent Scientific Corporation, Torrington, CT) by placing the animal in a holding tube with the pressure cuff at the base of the animal's tail. Systolic blood pressure (SBP), diastolic blood pressure (DBP), mean arterial pressure (MAP), and heart rate (HR) were measured in conscious rats at the end of each week. The mean of 10 consecutive readings was used for the reported values of SBP, DBP, MAP, and HR for each rat.

### Oral glucose tolerance test (OGTT)

Following 10 days after the osmotic minipump implantation, an oral glucose tolerance test was performed to determine insulin sensitivity at the whole-body level. On the evening (06:00 p.m.) of the day before the test, each rat was food-restricted to 4 g of chow and the wheels were locked. The next morning (08:00 – 09:00 a.m.), 0.5 ml of tail blood was collected before and 15, 30, 60, and 120 min after glucose feeding (1 g/kg BW) by gavage. Blood samples were mixed with EDTA as an anticoagulant, and plasma samples were centrifuged at 13,000 g at 4°C for 1 min. Plasma was kept at −80°C and used to determine the levels of glucose by colorimetric assay (Gesellschaft fur Biochemica and Dianostica, Wiesbadan, Germany) and insulin by radioimmunoassay (Linco Research, St. Charles, MO). Immediately after the test, each animal was subcutaneously given 2.5 ml of sterile 0.9% saline to replace fluid loss.

### Insulin action on skeletal muscle glucose uptake

Four days after the OGTT, animals were terminated to collect tissues and blood samples. Running wheels were locked approximately 24 h before the experiment. On the evening (18:00 h) of the day before the test, each rat was food-restricted to 4 g of chow. The next morning, animals were weighed and anesthetized with an intraperitoneal injection of thiopental (100 mg/kg body weight). Soleus muscles were isolated and prepared for *in vitro* incubation. The two soleus muscles were divided into three strips each. Two non-incubated soleus strips were quickly frozen in liquid nitrogen for subsequent analyses of signaling proteins. Four fresh soleus strips (~25 mg) were incubated for 30 min at 37°C in 3 ml of oxygenated Krebs-Henseleit buffer (KHB) supplemented with 8 mM D-glucose, 32 mM D-mannitol, and 0.1% radioimmunoassay-grade bovine serum albumin (Sigma Chemical, St. Louis, MO). Two of these four muscle strips were incubated in the absence of insulin and the other two were incubated in the presence of a maximally effective concentration of insulin (2 mU/ml; Human R, Eli Lilly, Indianapolis, IN). The flasks were continuously gassed with a mixture of 95% O_2_ and 5% CO_2_ throughout the incubation and glucose transport assay. After the first incubation period, two of the soleus strips (one incubated without insulin and one incubated with insulin) were removed, trimmed of fat and connective tissue, and quickly frozen in liquid nitrogen. These strips were subsequently used for the determination of signaling proteins in response to insulin activation by immunoblotting. The remaining muscle strips were rinsed for 10 min at 37°C in 3 ml of oxygenated KHB containing 40 mM D-mannitol, 0.1% BSA, and insulin, if previously present. Muscle strips were incubated for 20 min at 37°C in 2 ml of KHB containing 1 mM 2-[1,2-^3^H]deoxyglucose (2-DG, 300 μCi/mmol; PerkinElmer Life Sciences, Boston, MA), 39 mM [U-^14^C]mannitol (0.8 μCi/mmol; PerkinElmer Life Sciences), 0.1% BSA, and insulin, if previously present. At the end of the incubation period, the muscle strips were removed, trimmed of excess fat and connective tissue, and immediately frozen in liquid nitrogen and weighed. The frozen muscles were solubilized in 0.5 ml of 0.5 N NaOH and 10 ml of scintillation cocktail (Ultima Gold™; PerkinElmer Life Sciences) was added. The specific intracellular accumulation of 2-DG was determined as previously described using mannitol to correct for the extracellular accumulation of 2-DG (Henriksen and Halseth, 1994). Glucose transport activity was measured as the intracellular accumulation of 2-DG (in pmol/mg muscle wet weight/20 min).

### Tissue and blood collection

After the removal of muscle tissues to determine glucose transport activity, blood was collected from the abdominal vein. Whole blood was allowed to clot and was then centrifuged at 3,000 g for 20 min at 4°C to obtain serum, which was used to determine serum triglyceride levels. Immediately after blood collection, intra-abdominal fat and the heart were collected and weighed.

### Analyses of signaling molecules in skeletal muscle

Muscles were homogenized in ice-cold lysis buffer: 50 mM HEPES (pH 7.4), 150 mM NaCl, 1 mM CaCl_2_, 1 mM MgCl_2_, 2 mM EDTA, 10 mM NaF, 20 mM sodium pyrophosphate, 20 mM β-glycerophosphate, 10% glycerol, 1% Triton X-100, 2 mM Na_3_VO_4_, 10 μg/ml aprotinin and leupeptin, and 2 mM PMSF. After a 20-min incubation on ice, the homogenates were centrifuged at 13,000 g for 20 min at 4°C. Aliquots of supernatant were frozen at −80°C, and a portion of these homogenates was used to determine total protein content (BCA method, Sigma Chemical). Proteins in the homogenates were separated on 8 or 10% polyacrylamide gels and transferred onto nitrocellulose paper. Protein blots of samples from incubated and non-incubated muscles were probed with the appropriate dilution of commercially available antibodies against phospho-insulin receptor (IR)/IGF1R (Tyr^1158^/Tyr^1162^/Tyr^1163^) (Millipore, Billerica, MA), insulin receptor beta (4B8), phospho-IRS-1 (Ser^307^), IRS-1, phospho-Akt (Ser^473^), Akt, phospho-SAPK/JNK (Thr^183^/Tyr^185^), SAPK/JNK, phospho-p38 MAPK (Thr^180^/Tyr^182^), and p38 MAPK. Protein blots from non-incubated muscles were also probed with commercially available antibodies against GLUT-4, GLUT-1 (Santa Cruz Biotechnology, Santa Cruz, CA), phospho-AMPK Thr^172^, AMPK, ACE1 (Abcam Inc., Cambridge, MA), ACE2 (Millipore, Billerica, MA), AT1 receptor (Santa Cruz Biotechnology, Santa Cruz, CA), AT2 receptor (Santa Cruz Biotechnology, Santa Cruz, CA), MAS receptor (Santa Cruz Biotechnology, Santa Cruz, CA), 4-HNE (Abcam Inc., Cambridge, MA) and GAPDH. Subsequently, all blots were incubated with goat anti-rabbit, anti-mouse or anti-goat secondary antibodies conjugated with horseradish peroxidase (IgG-HRP). All antibodies, if not specified above, were purchased from Cell Signaling Technology (Beverly, MA). GAPDH was used as internal control to normalize signal intensities. Protein bands were visualized with enhanced chemiluminescence (PerkinElmer Life Sciences) on a C-Digit Blot Scanner (LI-COR Biotechnology, Lincoln, NE) with Image Studio Software version 3.1 for the quantitative analysis of band intensities.

### Statistical analysis

Two-way analysis of variance (ANOVA) was used to examine the main effects of ANGII infusion (SHAM or ANGII), exercise (SED or VWR) and the ANGII infusion x exercise interaction, and Tukey's test was used for post hoc analysis to identify the source of significant variance (SigmaPlot version 12.0; Systat Software, San Jose, CA). Data lacking normal distribution and/or equal variance were mathematically transformed to achieve normality and equal variance prior to two-way ANOVA being applied. Pearson correlation was used to evaluate associations between measured outcomes. Data are presented as the means ± SE. A value of *P* < 0.05 was considered to be statistically significant.

## Results

### Effect of VWR on body weight, heart and fat weight and lipid profiles

**Figure 1:**
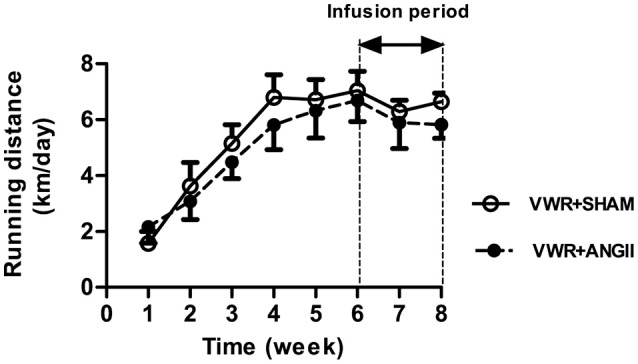
Running activity. Average daily voluntary running distances for SHAM and ANGII-infusion animals given access to VWR for 8 weeks. Values are presented as the means ± SE for 7–8 animals/group.

Figure 1 shows the pattern of running activity during the 8-week exercise period. No significant differences in running distances were observed between SHAM and ANGII groups throughout the experimental period. The running distances were gradually increased until reaching the peak distance during the 4- to 6-week period, averaged 7–8 km/day. Rats given access to VWR showed an 8–9% lower (*P* < 0.001) final body weight that was accompanied by decreased abdominal fat weight-to-body weight ratio (*P* < 0.001), however, the food and water intakes were significantly higher than the sedentary groups (Table 1). Moreover, the VWR group exhibited an enhanced heart weight-to-body weight ratio (*P* < 0.001) and a reduced total cholesterol (*P* < 0.001) and serum triglyceride levels (*P* < 0.05) compared to the sedentary group.

**Table 1:** Cardiometabolic parameters following SHAM or ANGII infusion with or without given access to VWR exercise.

**Parameters**	**SED**		**VWR**		**ANOVA effect of**		
	**SHAM**	**ANGII**	**SHAM**	**ANGII**	**ANGII**	**VWR**	ANGII^*^VWR
BW (g)	471 ± 11	468 ± 11	435 ± 10^*^	429 ± 9^†^	NS	*P* < 0.001	NS
HW/kg BW	2.65 ± 0.04	2.71 ± 0.06	3.17 ± 0.03^**^	3.22 ± 0.05^††^	NS	*P* < 0.001	NS
FW/kg BW	49.5 ± 3.3	47.0 ± 3.6	18.9 ± 1.1^**^	17.5 ± 1.2^††^	NS	*P* < 0.001	NS
Water intake (ml/day)	30 ± 1.1	31 ± 1.1	39 ± 1.7^**^	37 ± 1.3^†^	NS	*P* < 0.001	NS
Food intake (g/day)	20 ± 0.6	20 ± 0.4	25 ± 0.9^*^	24 ± 0.8^†^	NS	*P* < 0.05	NS
Fasting glucose, mg/dl	133 ± 5	136 ± 3	121 ± 2^*^	132 ± 5	NS	*P* < 0.05	NS
Fasting insulin, μU/ml	20.5 ± 4.0	21.5 ± 1.5	11.8 ± 0.8^*^	11.4 ± 0.8^††^	NS	*P* < 0.001	NS
HOMA-IR	6.59 ± 1.50	7.25 ± 0.62	3.57 ± 0.23^*^	3.90 ± 0.37^†^	NS	*P* < 0.001	NS
Total cholesterol (mg/dl)	82.8 ± 4.4	85.5 ± 3.3	74.5 ± 1.9^*^	68.5 ± 2.2^††^	NS	*P* < 0.001	NS
Triglyceride (mg/dl)	32.2 ± 3.1	33.3 ± 1.9	24.3 ± 1.9^*^	23.3 ± 3.3^†^	NS	*P* < 0.05	NS
Final heart rate, bpm	446 ± 16	460 ± 14	362 ± 6^**^	345 ± 7^††^	NS	*P* < 0.001	NS
Final SBP, mmHg	146 ± 2	192 ± 2^**^	137 ± 3^*^	175 ± 6^†^^§§^	*P* < 0.001	*P* < 0.05	NS
Final DBP, mmHg	108 ± 2	153 ± 7^**^	107 ± 2	141 ± 8^§§^	*P* < 0.001	NS	NS
Final MABP, mmHg	122 ± 4	167 ± 6^**^	119 ± 2	154 ± 7^§§^	*P* < 0.001	NS	NS

Values are the means± SE for 7–8 animals per group.BW indicates body weight; HW, heart weight; FW, fat weight; HOMA-IR, homeostasis model assessment-estimated insulin resistance; SBP, systolic blood pressure; DBP, diastolic blood pressure; MABP, mean arterial blood pressure.

* p < 0.05 vs. SED + SHAM group;

** p < 0.001 vs. SED + SHAM group;

† p <0.05 vs. SED + ANGII group;

†† p < 0.001 vs. SED + ANGII group;

§ p < 0.05 vs. VWR + SHAM group;

§§ *p < 0.001 vs. VWR + SHAM group*.

### Effects of VWR on heart rate and blood pressure

No significant changes in resting heart rate of the sedentary rats were observed along the experimental period. After 4 weeks of exercise sessions, the rats that were given access to VWR showed a gradual decrease in resting heart rate to approximately a 20% reduction (*P* < 0.001) by the end of the exercise period (Table 1). Both of the ANGII-infused groups had higher values of SBP, DBP and MABP (*P* < 0.001) when compared to the respective control groups. However, the extent of the increase in SBP due to ANGII infusion in VWR rats was lower than sedentary rats. In saline-infused groups, SBP was lower in VWR rats than (*P* < 0.05) sedentary rats.

### Effects of VWR on whole-body insulin sensitivity

**Figure 2:**
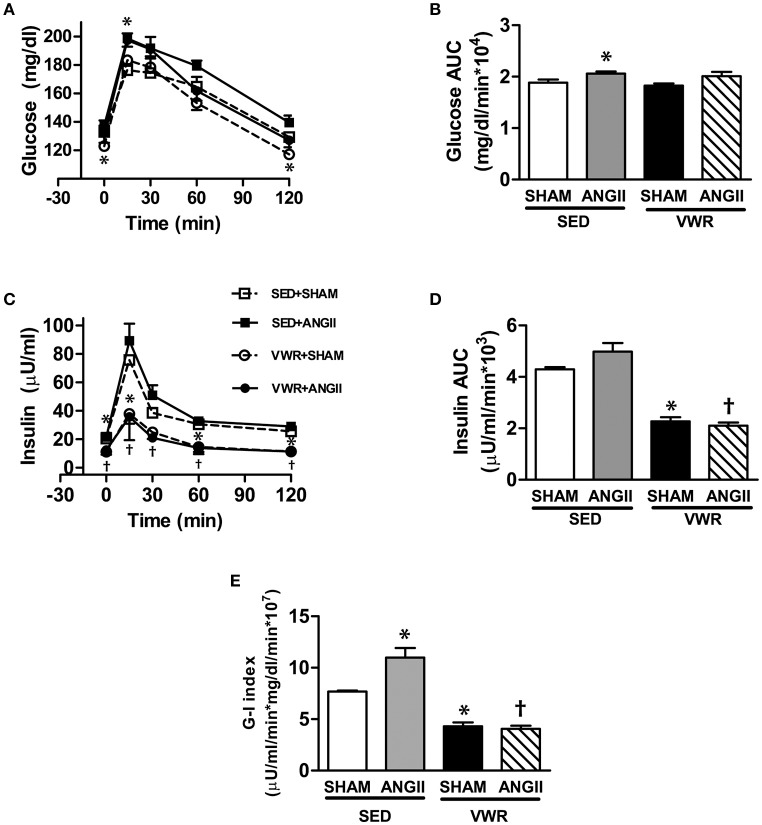
Glucose tolerance and insulin sensitivity after ANGII infusion with or without VWR. Plasma glucose **(A)** and plasma insulin **(C)** responses during OGTT and the glucose AUC **(B)**, insulin AUC **(D)**, and G–I index **(E)** were calculated to represent whole-body insulin sensitivity. Values are presented as the means ± SE for 7–8 animals/group. There was a significant main effect of exercise and ANGII infusion on the levels of plasma glucose **(A)**, the glucose AUC **(B)**, and G–I index **(E)**. A significant main effect of exercise was observed for the levels of plasma insulin **(C)** and the insulin AUC **(D)**. *Post hoc* analysis indicated *p < 0.05 vs. SED+SHAM group, ^†^*p* < 0.05 vs. SED+ANGII infusion group.

The profiles of plasma glucose and insulin levels during OGTT are shown in Figure 2. The plasma glucose level at the 15-min time points, the area under the curve (AUC) of glucose and the G-I index were significantly higher in the ANGII group when compared to the SHAM group. Contrary, rats given access to VWR demonstrated a significant decrease in fasting plasma glucose and insulin levels (Table 1) and significant decreases in plasma insulin levels at the 15-, 60- and 120-min time points and insulin AUC. With ANGII infusion, the levels of fasting plasma insulin and plasma insulin levels at the 15-, 30-, 60-, and 120-min time points and insulin AUC in VWR rats was lower than those of the sedentary rats. Thus, a significant decrease in G-I index (Figure 2E) and HOMA-IR (Table 1) clearly indicated that VWR effectively improved whole-body insulin sensitivity in both saline- and ANGII-infused rats.

### Effects of VWR on skeletal muscle glucose transport activities

**Figure 3:**
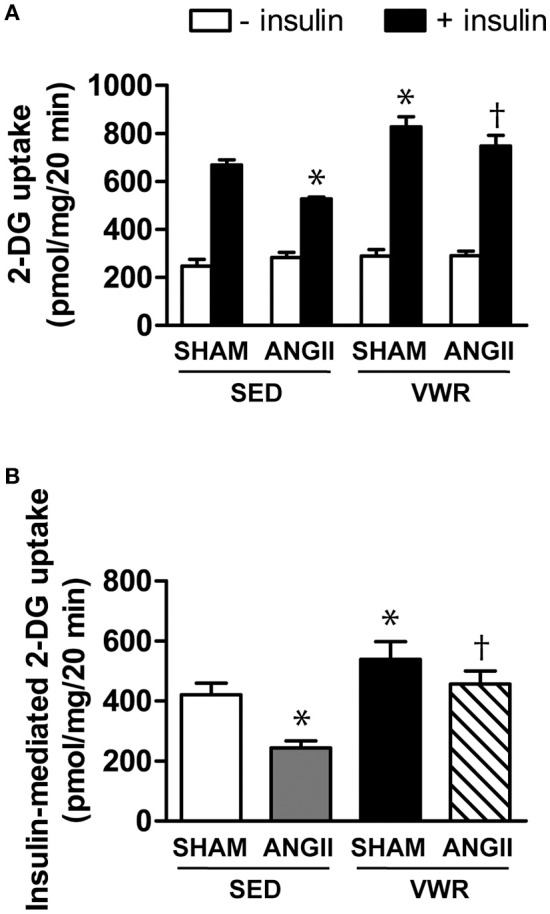
Effects of ANGII administration with or without VWR on soleus muscle glucose transport. *In vitro* rates of 2-DG uptake in the absence and presence of insulin (2 mU/ml) **(A)** and net increases above basal rates of 2-DG transport activities due to insulin **(B)** in soleus muscles of rats receiving normal saline infusion or ANGII infusion with or without VWR. Values are presented as the means ± SE for 7–8 animals/group. There was a significant main effect of exercise and ANGII infusion on 2-DG uptake **(A)** and insulin-mediated 2-DG uptake **(B)**. *Post hoc* analysis indicated *p < 0.05 vs. SED+SHAM group, ^†^*p* < 0.05 vs. SED+ANGII infusion group.

Glucose uptake in the soleus muscle without or with insulin stimulation and insulin-mediated glucose above basal levels are demonstrated in Figures 3A,B, respectively. When compared to SHAM, chronic ANGII infusion brought about significant reductions in insulin-stimulated and insulin-mediated glucose uptake by 21 and 42%, respectively. In contrast, VWR enhanced insulin-stimulated and insulin-mediated glucose uptake by 24 and 28% (*P* < 0.05), respectively. Importantly, rats given access to VWR prior to receiving ANGII infusion exhibited significant increases in insulin-stimulated and insulin-mediated glucose uptake compared to sedentary rats with ANGII infusion. Therefore, regular exercise prevented ANGII-induced insulin resistance of skeletal muscle glucose uptake.

### Effects of VWR on insulin signaling molecules and GLUT-1 and GLUT-4 abundance in skeletal muscle

**Figure 4:**
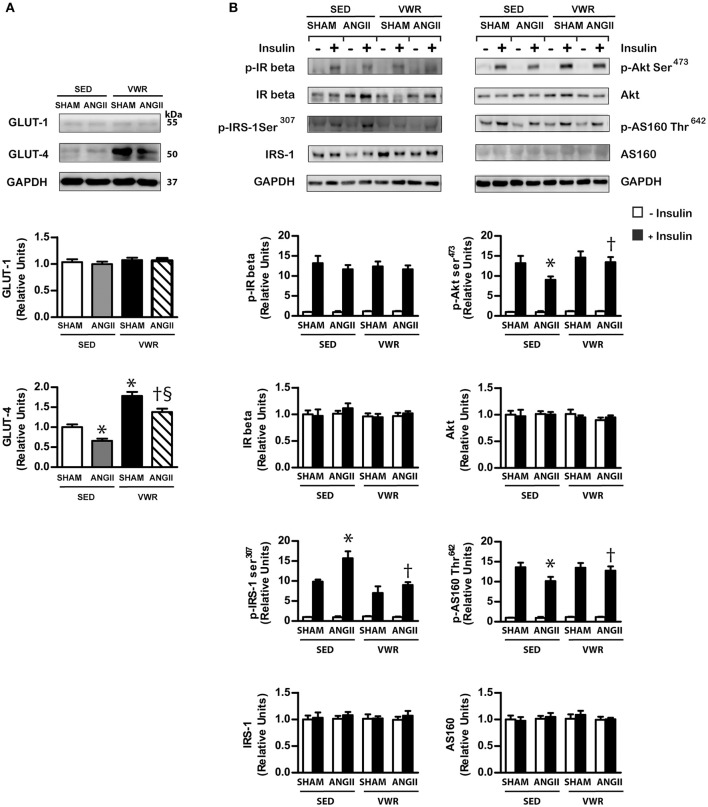
GLUT-1 and GLUT-4 abundance and insulin signaling molecules in skeletal muscle. GLUT-1 and GLUT-4 protein expression **(A)** in non-incubated soleus muscle that were obtained from rats receiving normal saline or ANGII infusion with or without VWR. Insulin-induced expression and phosphorylation of insulin signaling molecules in incubated soleus muscles in the absence or the presence of insulin (2 mU/ml) **(B)**. Proteins were determined by immunoblot analysis and were normalized to GAPDH. Data are presented as the fold change over the SED+SHAM group. Representative bands from the C-Digit Blot Scanner are displayed at the top of the figure. Values are presented as the means ± SE for 7–8 animals/group. There was a significant main effect of exercise and ANGII infusion on GLUT-4 abundance and the phosphorylation of insulin signaling proteins **(B)**. *Post hoc* analysis indicated *p < 0.05 vs. SED+SHAM group, ^†^*p* < 0.05 vs. SED+ANGII infusion group. ^§^*p* < 0.05 vs. VWR+SHAM group.

The expression and phosphorylation levels of IR-β, IRS-1, Akt and AS160 in the soleus muscle of both SHAM and ANGII-infused groups (Table 2) and the ability of insulin to activate these insulin signaling proteins in the SHAM group were not affected by VWR (Figure 4B). Rats given access to VWR prior to ANGII infusion did not show defects in the insulin signaling proteins as presented in the ANGII-infused sedentary rats. These results indicated that VWR did not affect the insulin signaling molecules in skeletal muscle of normal rats but it could prevent the defects of insulin signaling molecules induced by ANGII infusion. Furthermore, no significant differences in GLUT-1 protein expression were observed among groups (Figure 4A). While ANGII infusion suppressed GLUT-4 protein abundance (*P* < 0.05) in sedentary rats, VWR increased GLUT-4 abundance by 45 and 75% in ANGII- and saline-infused rats, respectively (Figure 4A).

**Table 2:** Expressions of insulin signaling protein in non-incubated soleus muscle.

**Insulin signaling**	**SED**		**VWR**		**Representative blot**
	**SHAM**	**ANGII**	**SHAM**	**ANGII**	
p-IRβ	1.00 ± 0.09	1.11 ± 0.12	1.15 ± 0.11	1.19 ± 0.15	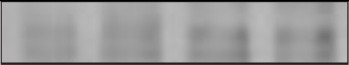
IRβ	1.00 ± 0.11	0.93 ± 0.11	0.95 ± 0.08	1.14 ± 0.07	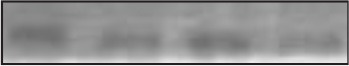
p-IRS1 Ser^307^	1.00 ± 0.08	1.21 ± 0.15	0.89 ± 0.17	0.93 ± 0.14	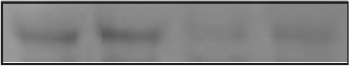
IRS1 Ser^307^	1.00 ± 0.06	1.04 ± 0.09	1.12 ± 0.08	1.26 ± 0.12	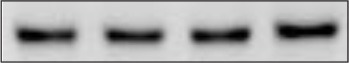
p-Akt Ser^473^	1.00 ± 0.01	1.09 ± 0.05	1.19 ± 0.07	1.14 ± 0.05	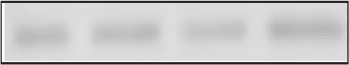
Akt Ser^473^	1.00 ± 0.07	0.98 ± 0.08	0.97 ± 0.07	0.91 ± 0.11	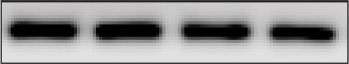
p-AS160 Thr^642^	1.00 ± 0.06	1.13 ± 0.11	1.11 ± 0.11	1.24 ± 0.13	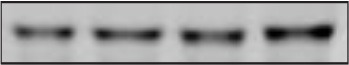
AS160 Thr^642^	1.00 ± 0.16	0.92 ± 0.17	1.25 ± 0.23	0.96 ± 0.10	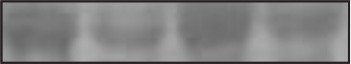

*Expressions of insulin signaling protein including IRβ (IR beta), IRβ(Tyr^1158^/Tyr^1162^/Tyr^1163^) phosphorylation (p-IRβ), IRS-1, IRS-1 Ser^307^ phosphorylation (p-IRS1 Ser^307^), Akt, Akt Ser^473^ phosphorylation (p-Akt Ser^473^), AS160 and AS160 Thr^642^ phosphorylation (p-AS160 Thr^642^) in non-incubated soleus muscle of SHAM and ANGII infusion at 100 ng/kg/min with or without VWR*.

### Effects of VWR on ROS generation and p38 MAPK and SAPK/JNK

**Figure 5:**
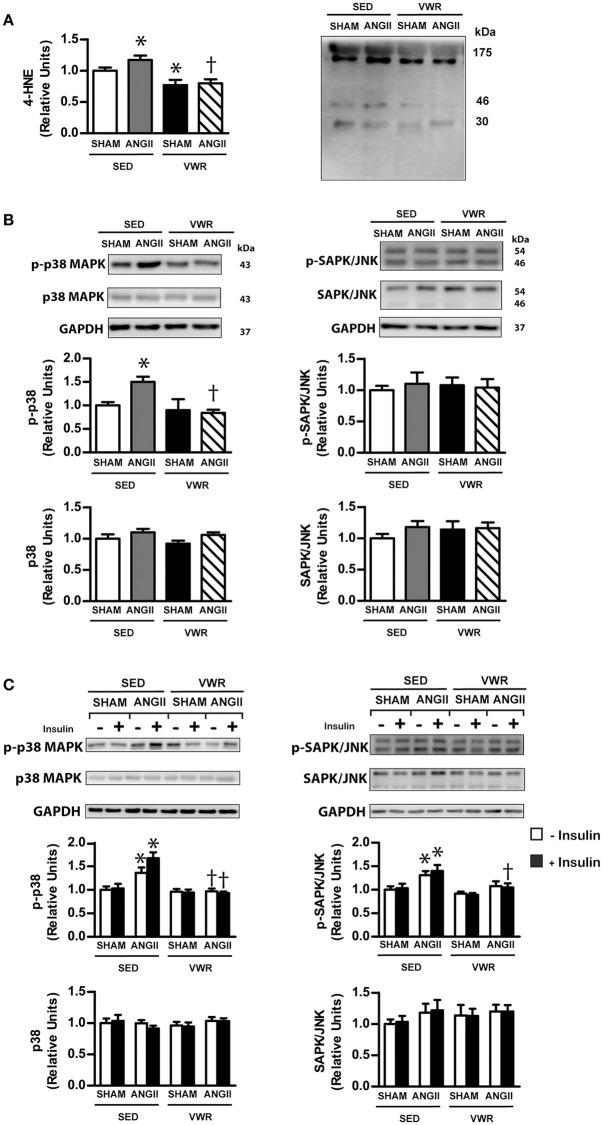
Effects of ANGII infusion with or without VWR on ROS generation and p38 MAPK pathway. 4-HNE, a marker of oxidative stress, is induced by ANGII infusion **(A)**. Expression of the signaling proteins p38 MAPK and phosphorylated p38 MAPK (Thr^180^/Tyr^182^) (p-p38 MAPK), SAPK/JNK and phosphorylated SAPK/JNK (Thr183/Tyr185) (p-SAPK/JNK), in non-incubated soleus muscle **(B)**. Insulin-induced expression of p38 MAPK and p-p38 MAPK, SAPK/JNK and p-SAPK/JNK in soleus muscles incubated in the absence or the presence of insulin (2 mU/ml) **(C)**. Proteins were determined by immunoblot analysis and were normalized to GAPDH. Data are presented as the fold change over the SED+SHAM group. Representative bands from the C-Digit Blot Scanner are displayed at the right side (4-HNE) and at the top of the figure. Values are presented as the means ± SE for 7–8 animals/group. There was a significant main effect of exercise and ANGII infusion on 4-HNE **(A)** and the phosphorylation of p38 MAPK in non-incubated and incubated soleus muscle **(B)**. *Post hoc* analysis indicated *p < 0.05 vs. SED+SHAM group, ^†^*p* < 0.05 vs. SED+ANGII infusion group.

4-hydroxynonenal (4-HNE) is a product of lipid peroxidation and has been used as an indicator of oxidative stress (Browning and Horton, 2004; Wei Sun et al., 2008). We found that ANGII infusion significantly increased the levels of 4-HNE and phosphorylated p38 MAPK, as well as the phosphorylation levels of p38 MAPK and SAPK/JNK under insulin-stimulated conditions in the soleus muscle (Figure 5). Interestingly, increases in 4-HNE and the activities of p38 and SAPK/JNK due to ANGII infusion did not occur in rats given access to VWR (Figure 5 and Supplementary Material). These experiments demonstrated that VWR can attenuate the levels of oxidative stress and the activities of stress kinases, induced by ANGII, in the soleus muscle.

### Effect of VWR on the expression levels of AMPK and RAS protein in skeletal muscle

**Figure 6:**
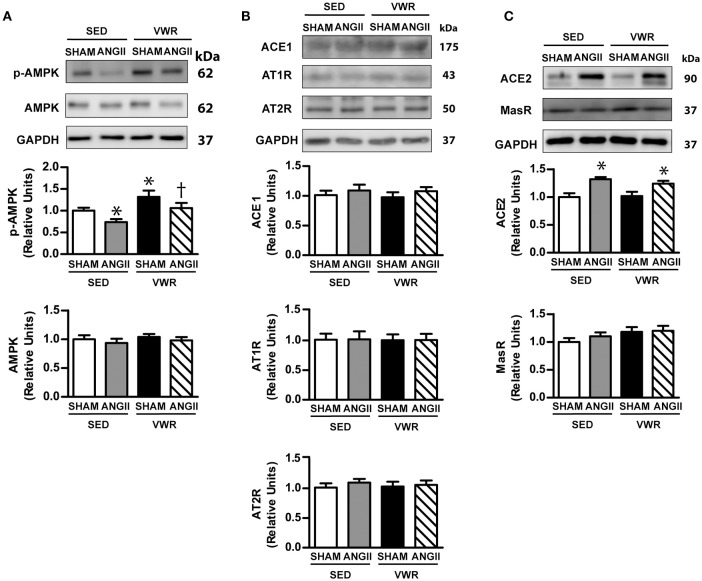
Effects of ANGII infusion with or without VWR on AMPK, ACE1, ACE2, AT1R, AT2R, and MAS receptor in skeletal muscle. Effects of ANGII infusion on the expression of AMPK and phosphorylated AMPK Thr^172^ (p-AMPK Thr^172^) **(A)** and ACE1, AT1R and AT2R **(B)** and ACE2 and MAS receptor (MasR) **(C)** in non-incubated soleus muscle. Proteins were determined by immunoblot analysis and were normalized to GAPDH. Data are presented as the fold change over the SED+SHAM group. Representative bands from the C-Digit Blot Scanner are displayed at the top of the figure. Values are presented as the means ± SE for 7–8 animals/group. There was a significant main effect of exercise and ANGII infusion on the phosphorylation of AMPKThr^172^ (p-AMPK Thr^172^) **(A)**. A significant main effect of ANGII infusion was observed on ACE2 **(C)**. *Post hoc* analysis indicated *p < 0.05 vs. SED+SHAM group, ^†^*p* < 0.05 vs. SED+ANGII infusion group.

AMPK and proteins in the alternative RAS system, ACE2/ANG-(1-7)/MasR axis, can modulate glucose transport activity in skeletal muscle in an insulin-independent manner. We found that VWR enhanced the phosphorylation level of AMPK Thr^172^ in the soleus muscle of saline-infused rats (Figure 6A), while the impaired phosphorylation level of AMPK Thr^172^ induced by ANGII infusion was not observed in the soleus muscle of rats that regularly exercised prior to ANGII infusion. ANGII infusion significantly enhanced the expression level of ACE2 but not other molecules. In addition, VWR did not modulate the expression levels of other proteins in the RAS system including ACE1, ACE2, MasR, AT1R, and AT2R (Figures 6B,C).

### Correlations between abdominal fat accumulation and insulin sensitivity

**Figure 7:**
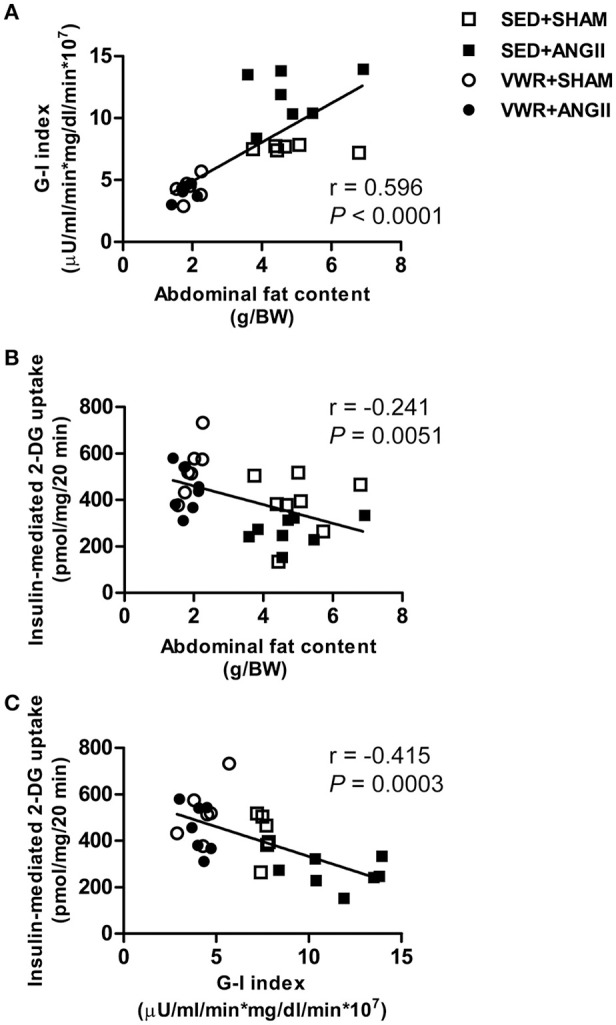
Correlations between abdominal fat content and insulin sensitivity. Correlation between G-I index and abdominal fat content **(A)** and correlation between insulin-mediated 2-DG uptake in soleus muscle and abdominal fat content **(B)** and G-I index **(C)**. Pearson's correlation was used to analyze the correlation between measured outcomes.

Figure 7 demonstrates the correlation between G-I index and abdominal fat content (Figure 7A), insulin-mediated 2-DG uptake and abdominal fat content (Figure 7B) and insulin-mediated 2-DG uptake and G-I index (Figure 7C). An improvement in whole-body insulin sensitivity was associated with an increase in insulin sensitivity in skeletal muscle. In addition, an improvement of insulin sensitivity at the whole-body and skeletal muscle levels was associated with a reduction in abdominal fat content.

## Discussion

Previous studies have demonstrated a broad range of distances achieved by rodents on running wheels. For example, low activity or aging animals voluntarily ran only 1–2 km/day (Wei Sun et al., 2008; Garvey et al., 2015), whereas rodents that were genetically selected for high wheel-running activity ran up to 10–15 km/day (Houle-leroy et al., 2000; Dumke et al., 2001). The average running distances monitored in this experiment were ~ 5–6 km/day, which is comparable to previously reported distances (Ishizawa et al., 2001; Kinnick et al., 2002; Lemieux et al., 2005). The main finding of this study provided new data revealing that voluntary exercise at this level is sufficient to prevent the progression of insulin resistance of glucose transport and to impair insulin signaling molecules in skeletal muscle of ANGII-infused rats. More importantly, we demonstrated that skeletal muscle of ANGII-infused rats that underwent VWR exhibited increases in GLUT-4 protein expression and the phosphorylation level of AMPK Thr^172^ and exhibited decreases in the oxidative stress marker and the phosphorylation levels of p38 MAPK and SAPK/JNK. Nevertheless, this adaptive response in muscle occurred independent of significant changes in protein expression of the renin-angiotensin system, including AT1R, AT2R, ACE, ACE2, and MasR. Moreover, VWR attenuated multiple cardiometabolic parameters, including reduced body weight, fat weight, and lipid profiles, as well as decreased heart rate and systolic blood pressure. Therefore, this investigation illustrated that voluntary exercise is an effective strategy that protects against insulin resistance of skeletal muscle glucose transport and prevents the development of cardiometabolic risks induced by chronic ANGII administration.

ANGII plays an essential role in various physiological functions, and overactivation of the RAS is associated with several pathological conditions such as essential hypertension and insulin resistance (Henriksen et al., 2001; Ran et al., 2004; Olivares-Reyes et al., 2009; Chen et al., 2010; Luther and Brown, 2011; Deji et al., 2012). Oxidative stress has been suggested as a link between ANGII and insulin resistance. For instance, *in vitro* studies using L6 myotube (Wei et al., 2006, 2008) and isolated muscle (Diamond-Stanic and Henriksen, 2010) indicated that ANGII activated oxidative stress through NADPH oxidase, which impaired insulin signaling molecules, including decreasing the phosphorylation levels of IRS-1, Akt and GLUT-4 translocation to plasma membrane and reduced glucose uptake. In addition to the direct effect of oxidative stress on insulin signaling transduction, oxidative stress also stimulated other transcription genes or stress kinases such as NF-κB, TNF-α, and MAPK, which impaired the engagement of insulin signaling cascade. Consistent with our previous report (Surapongchai et al., 2017), the present study demonstrated that ANGII infusion at 100 ng/kg/min enhanced oxidative stress marker and concomitantly increased the activity of p38 MAPK and SAPK/JNK and insulin resistance of skeletal muscle with multiple post-receptor defects. Importantly, we presented new data showing that ANGII-induced insulin resistance of skeletal muscle and elevated level of oxidative stress marker did not develop in VWR rats. The phosphorylation levels of insulin-stimulated IRS-1 Ser^307^, Akt Ser^473^, and AS160 Thr^642^ and the activity of p38 MAPK and SAPK/JNK in ANGII-infused rats, when given access to running wheels, were normalized to the levels of the saline infusion group. Furthermore, we found that ANGII infusion did not decrease AMPK activity and GLUT-4 abundance in skeletal muscle of VWR rats. Thus, our observations supported the notion that voluntary exercise protects against the development of skeletal muscle insulin resistance in ANGII-infused rats partly by relieving oxidative stress formation and MAPK activities and by preservation of GLUT-4 and AMPK activity.

Numerous studies have reported that activation of proteins in the novel RAS pathway through the ACE2/ANG-(1-7)/MasR axis can improve glucose metabolism (Prasannarong et al., 2012; Santos et al., 2013; Santos and Andrade, 2014). In the present study, we observed that the levels of AT1R, AT2R, ACE1, ACE2, and MasR proteins were not affected by VWR. Our observations indicated that the mechanism by which voluntary exercise protected against ANGII-induced insulin resistance of skeletal muscle glucose transport was independent of modifications in protein expression of the RAS pathways. As adaptations to exercise training could be specific and vary according to the training regime (Martinez-Valdes et al., 2017), we speculated that the differential effects of endurance exercise training and voluntary exercise on proteins in the RAS pathways might be due to differences in the pattern of muscle contraction (continuous vs. intermittent bursts), load intensity and exercise volume (Frantz et al., 2017).

Increasing evidence revealed that abdominal adiposity can produce pro-inflammatory cytokines and oxidative stress, which stimulate stress kinases and disturb insulin action in skeletal muscle, liver and vascular tissue (Shoelson et al., 2006; Rains and Jain, 2011). In this study, although rats given access to VWR consumed more food, the abdominal fat weight-to-body weight (FW/BW) ratio of these rats was approximately 60% lower than the sedentary rats. Interestingly, we observed that the FW/BW ratio was positively correlated (*P* < 0.001, *r* = 0.596) with the G-I index (lower values represent higher whole-body insulin sensitivity) and was inversely correlated (*P* < 0.05, *r* = −0.241) with insulin-mediated skeletal muscle glucose uptake. Thus, our results support the concept that the favorable metabolic effects of voluntary exercise on glucose metabolism at the whole-body and skeletal muscle levels could be mediated through a reduction in abdominal fat accumulation in both ANGII-infused and SHAM rats. Apart from the metabolic effects, our findings demonstrate that VWR can attenuate risks of cardiovascular diseases such as significant reductions in systolic blood pressure, serum levels of triglyceride and cholesterol with a trend to lower DBP and MABP. It has been recognized that exercise training induces several adaptions including modifications of the autonomic nervous system. For example, an increase in sympathetic activity enhances lipolysis from adipocytes, whereas a reduction in heart rate at rest occurs partly as a result of an increase in the parasympathetic tone to the heart (Messina et al., 2013, 2017). In this study, a significant decrease in resting heart rate, systolic blood pressure and abdominal fat content was observed in rats given access to running wheel. Thus, our observations are consistent with the notion that the adaptive changes in the autonomic nervous system could contribute to the preventive effects of VWR on the development of cardiometabolic risks induced by chronic ANGII infusion.

## Conclusion

Our findings underscore the benefits of an active lifestyle for the prevention of cardiometabolic risks induced by chronic infusion with ANGII. We demonstrated that prior exercise by VWR effectively protected against insulin resistance of glucose transport and impaired insulin signaling molecules in the soleus muscle, which was associated with a significant increase in AMPK Thr^172^ and decreases in oxidative stress and insulin-induced activities of MAPKs without significant changes in expression of proteins in the RAS pathways. Moreover, we found that an improvement in whole-body insulin sensitivity positively correlated with an enhancement of skeletal muscle glucose disposal. Enhancements of whole body and skeletal muscle insulin action inversely correlated with the amount of abdominal fat accumulation.

## Author contributions

JS and VS: Conception and design of experiments; JS, YR, JB, and VS: Performance of experiments; JS and VS: Data analysis; JS, YR, JB, and VS: Preparation and approval of final manuscript.

### Conflict of interest statement

The authors declare that the research was conducted in the absence of any commercial or financial relationships that could be construed as a potential conflict of interest.
