# Supplementary material for: Exercise Protects Against Defective Insulin Signaling and Insulin Resistance of Glucose Transport in Skeletal Muscle of Angiotensin II-Infused Rat
Source: Front Physiol. 2018 Apr 11;9:358. doi: 10.3389/fphys.2018.00358 (PMC5904253; doi:10.3389/fphys.2018.00358)
Supplement: Supplementary file 1 [file Image1.PDF]

Supplementary material

1. Original gels of expressions of insulin signaling protein in non-incubated soleus muscle represented in Table 2

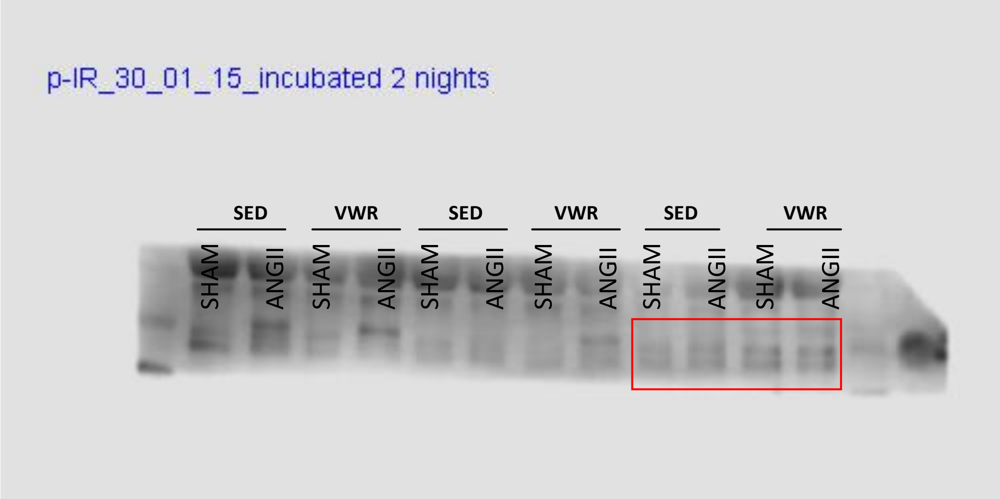

Figure 1.1 Expressions of IRβ(Tyr^{1158}/Tyr^{1162}/Tyr^{1163}) phosphorylation (p-IRβ) in non-incubated soleus muscle

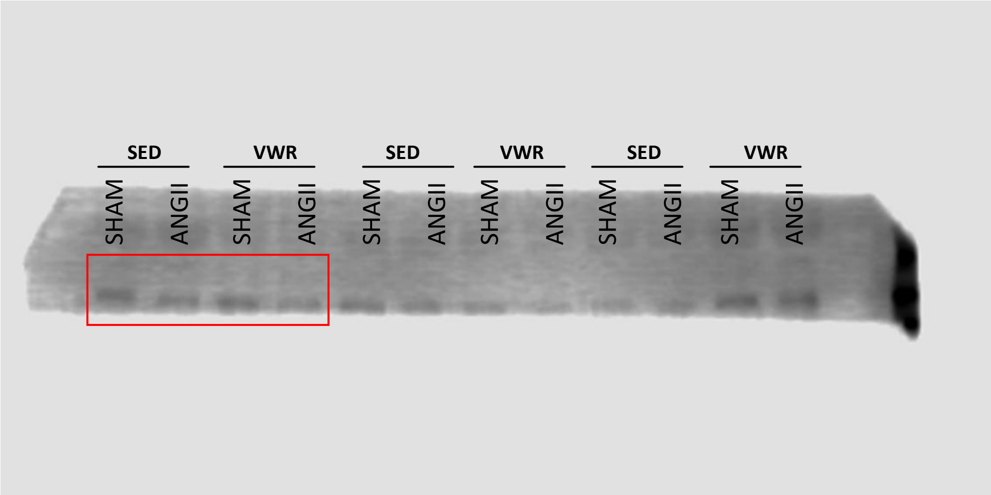

Figure 1.2 Expressions of IRβ in non-incubated soleus muscle

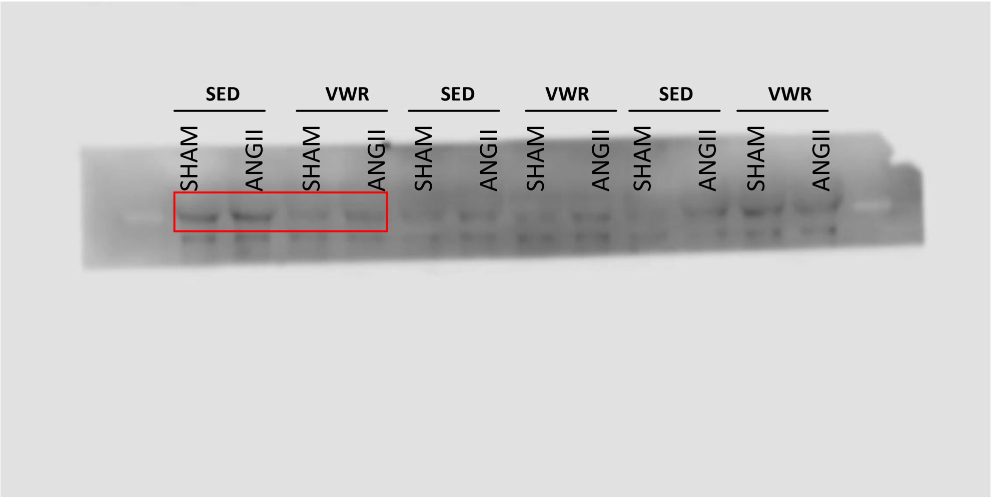

Figure 1.3 Expressions of IRS-1 Ser^{307} phosphorylation (p-IRS1 Ser^{307}) in non-incubated soleus muscle

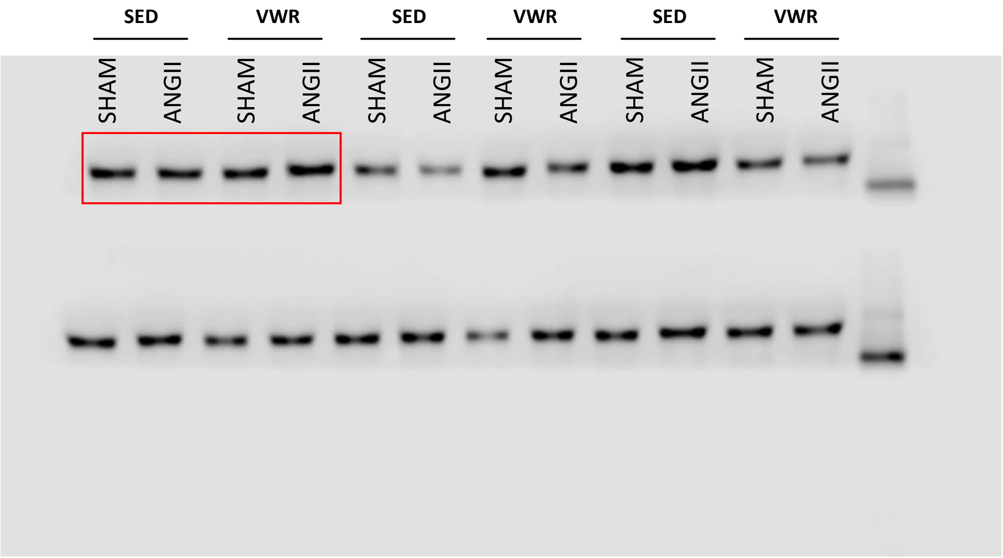

Figure 1.4 Expressions of IRS-1 in non-incubated soleus muscle

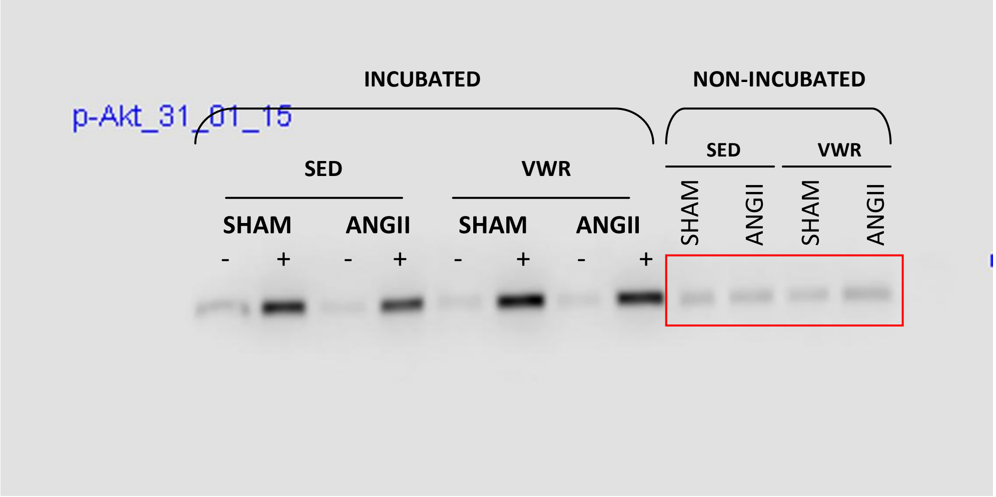

Figure 1.5 Expressions of Akt Ser^{473} phosphorylation (p-Akt Ser^{473}) in non-incubated soleus muscle

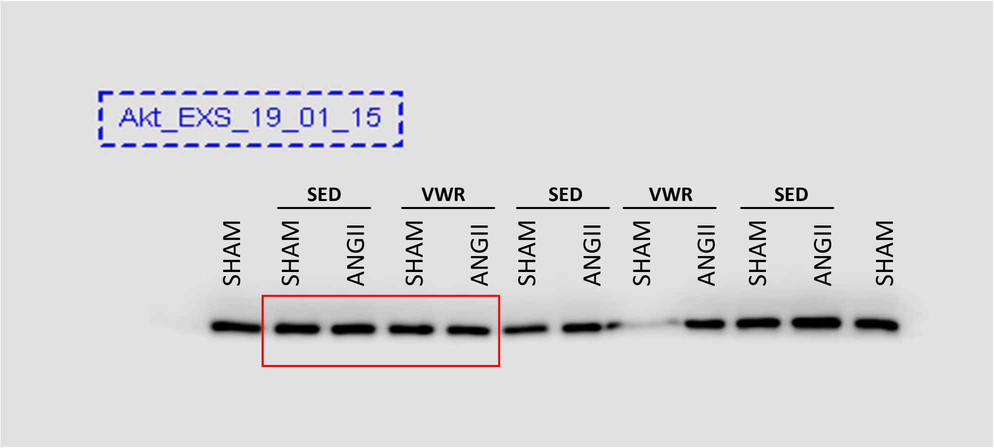

Figure 1.6 Expressions of Akt in non-incubated soleus muscle

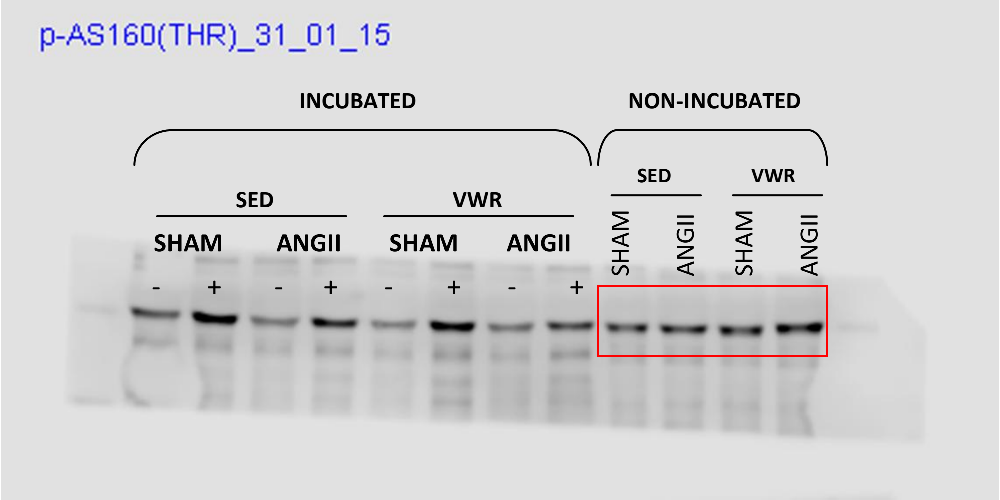

Figure 1.7 Expressions of AS160 Thr^{642} phosphorylation (p-AS160 Thr^{642}) in non-incubated soleus muscle

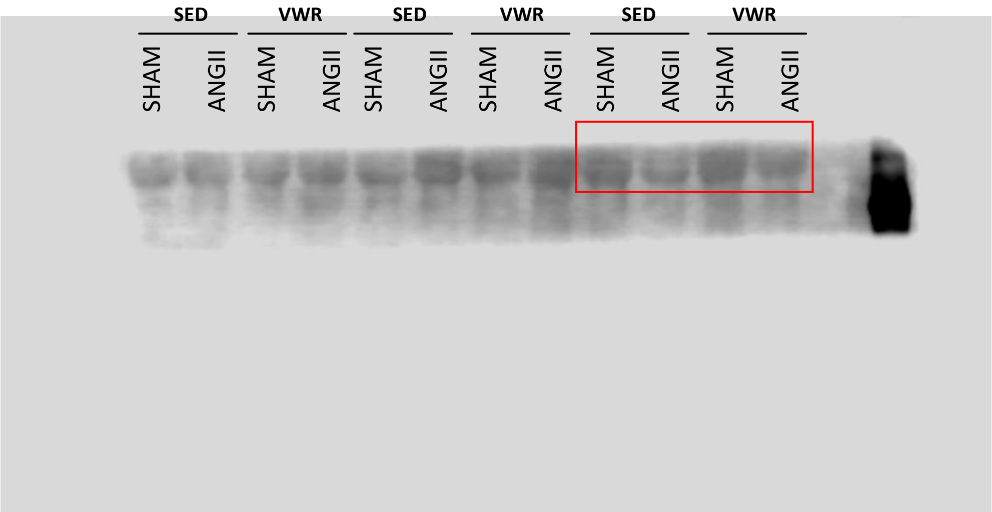

Figure 1.8 Expressions of AS160 in non-incubated soleus muscle

2. Original gels of expressions of GLUT-1 and GULT-4 protein in non-incubated soleus muscle represented in Figure 4A and insulin signaling protein in incubated soleus muscle represented in Figure 4B

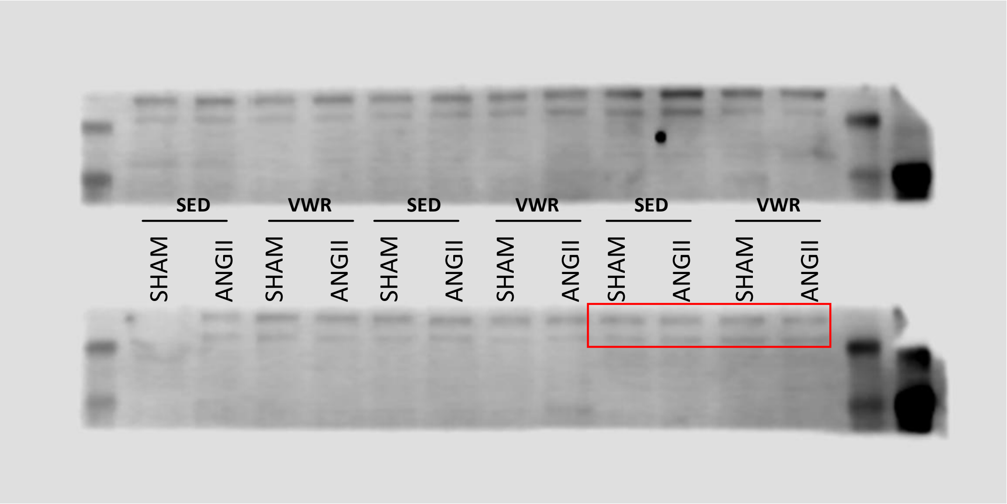

Figure 2.1 Expressions of GLUT-1 in non-incubated soleus muscle

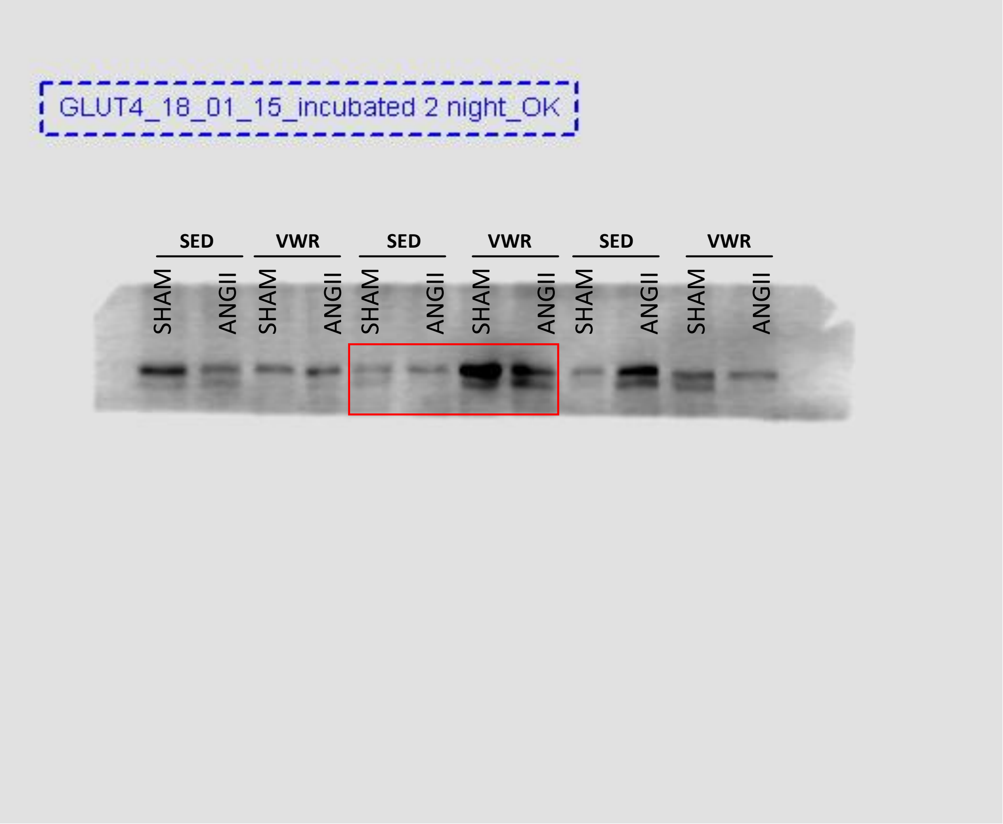

Figure 2.2 Expressions of GLUT-4 in non-incubated soleus muscle

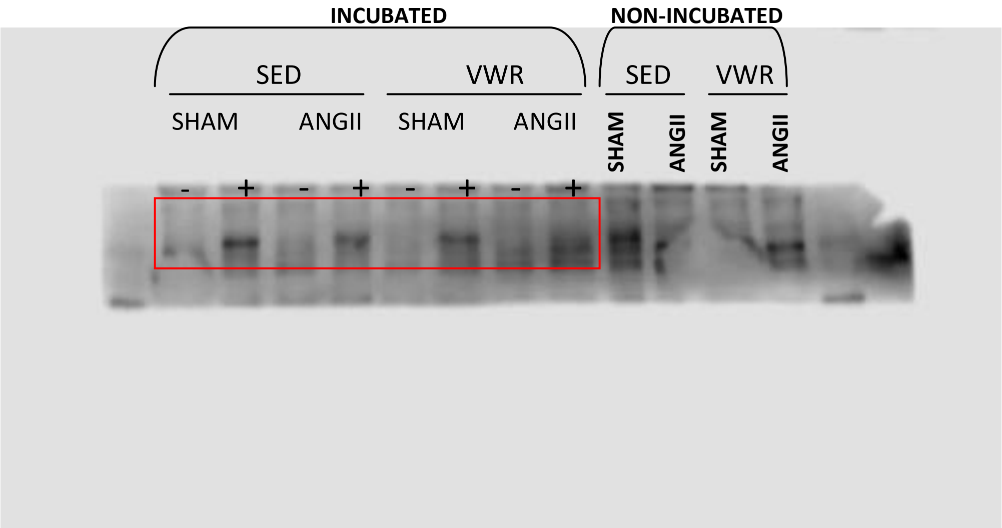

Figure 2.3 Expressions of IRβ(Tyr^{1158}/Tyr^{1162}/Tyr^{1163}) phosphorylation (p-IRβ) in incubated soleus muscle

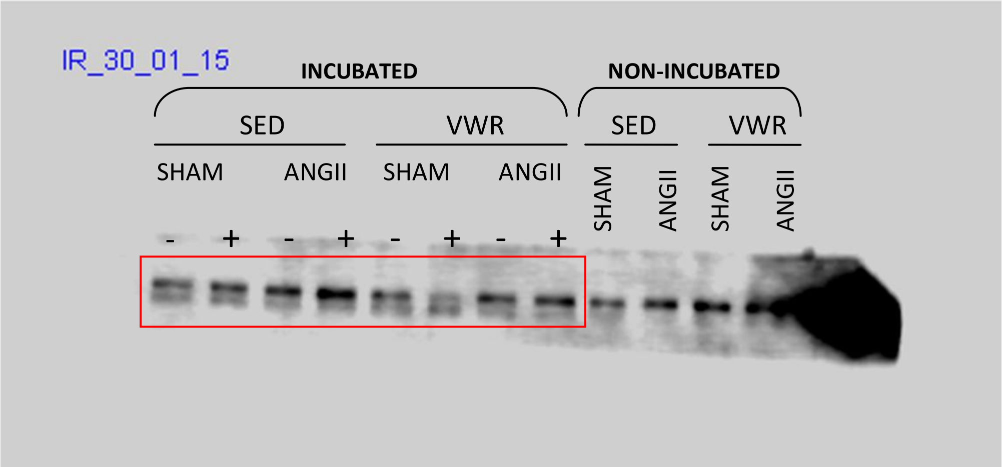

Figure 2.4 Expressions of IRβ in incubated soleus muscle

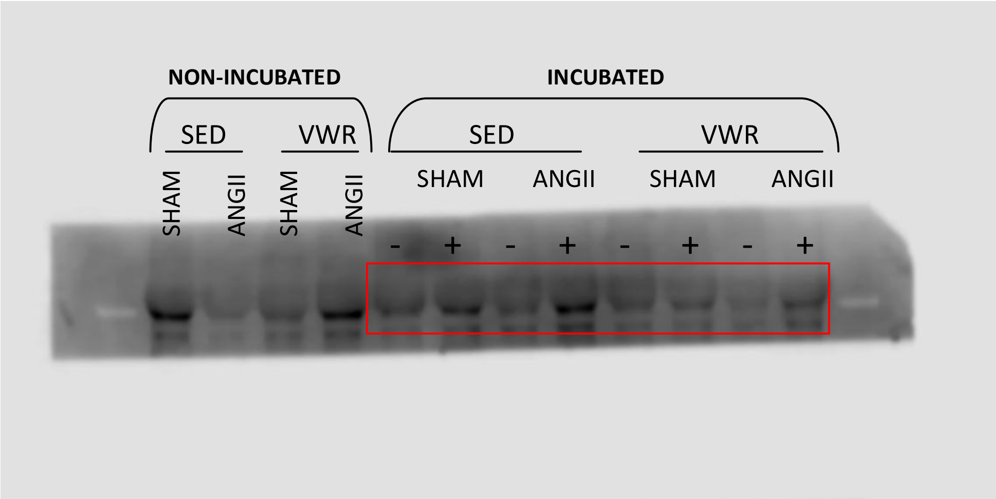

Figure 2.5 Expressions of IRS-1 Ser^{307} phosphorylation (p-IRS1 Ser^{307}) in incubated soleus muscle

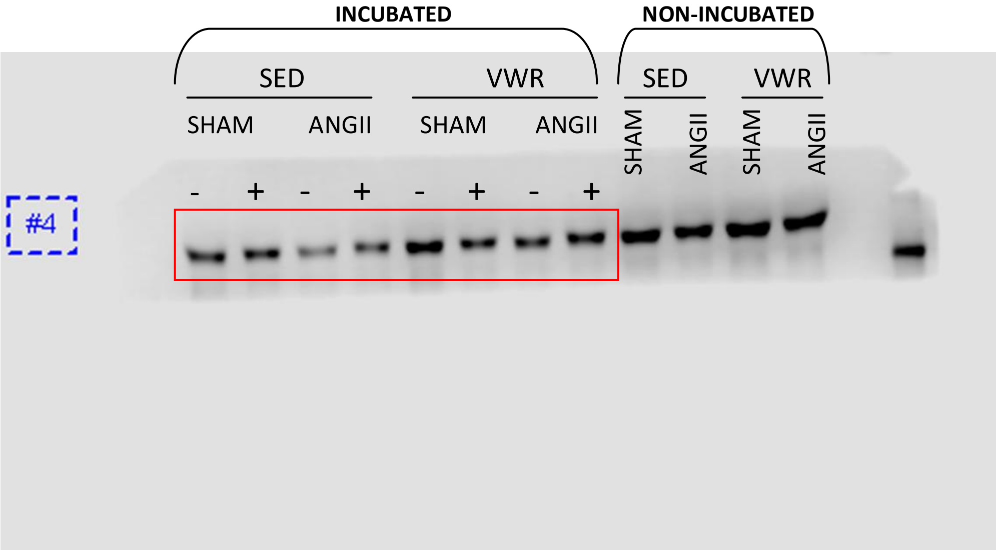

Figure 2.6 Expressions of IRS-1 in incubated soleus muscle

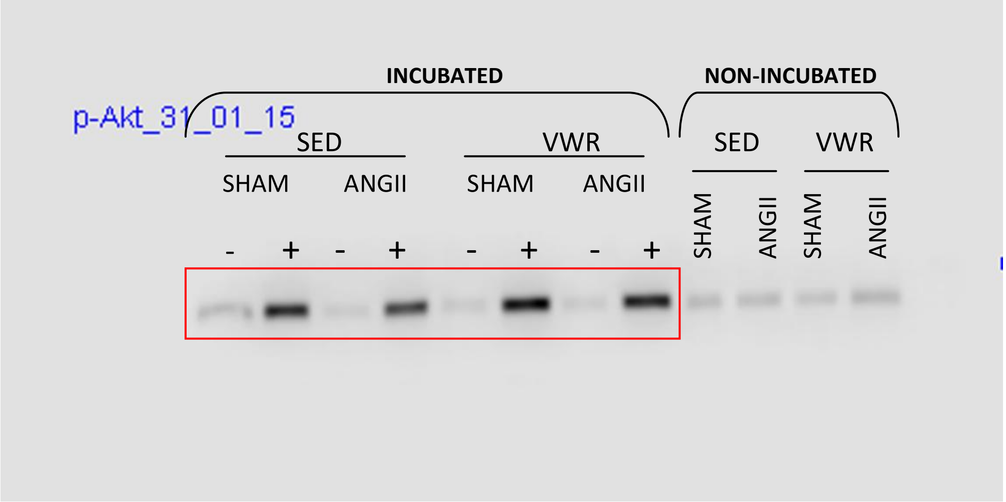

Figure 2.7 Expressions of Akt Ser473 phosphorylation (p-Akt Ser473) in incubated soleus muscle

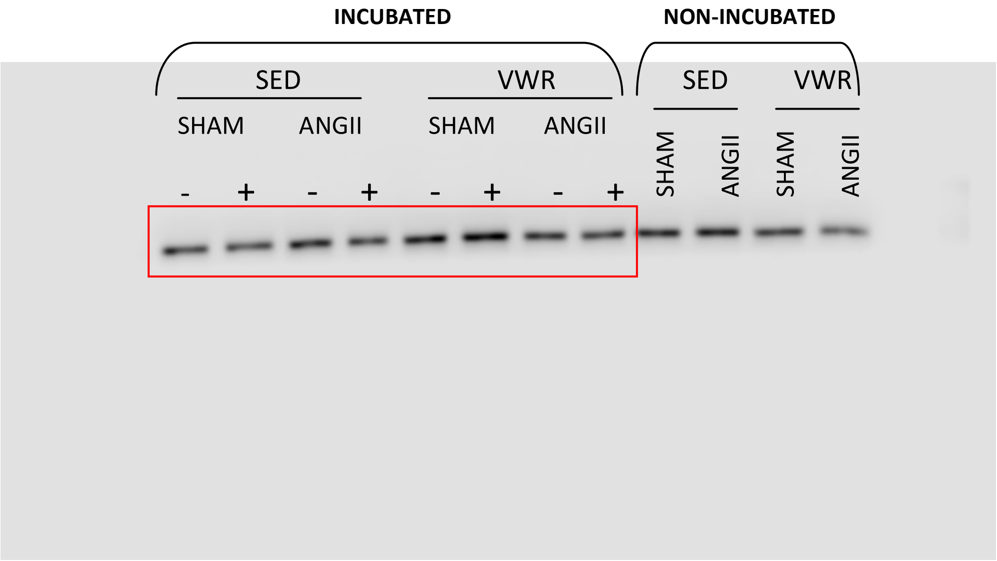

Figure 2.8 Expressions of Akt in incubated soleus muscle

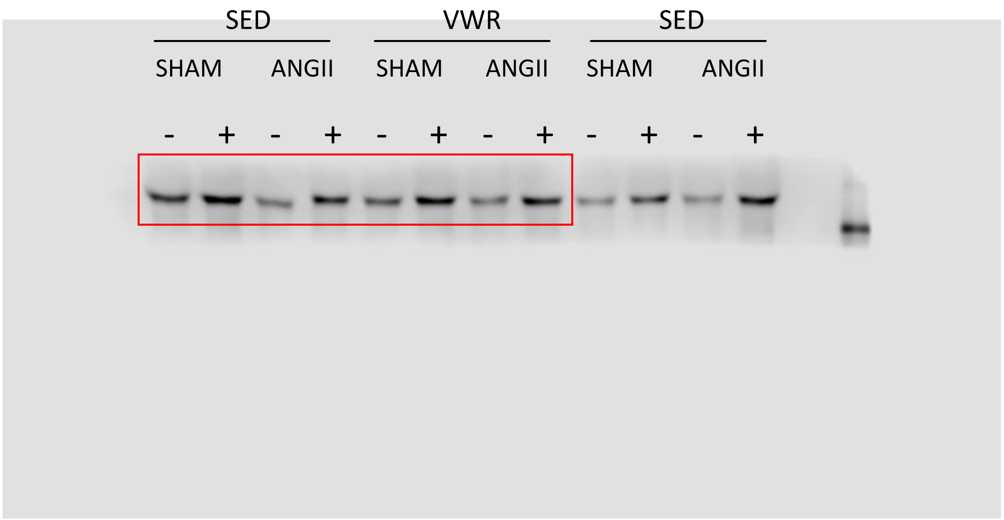

Figure 2.9 Expressions of AS160 Thr^{642} phosphorylation (p-AS160 Thr^{642}) in incubated soleus muscle

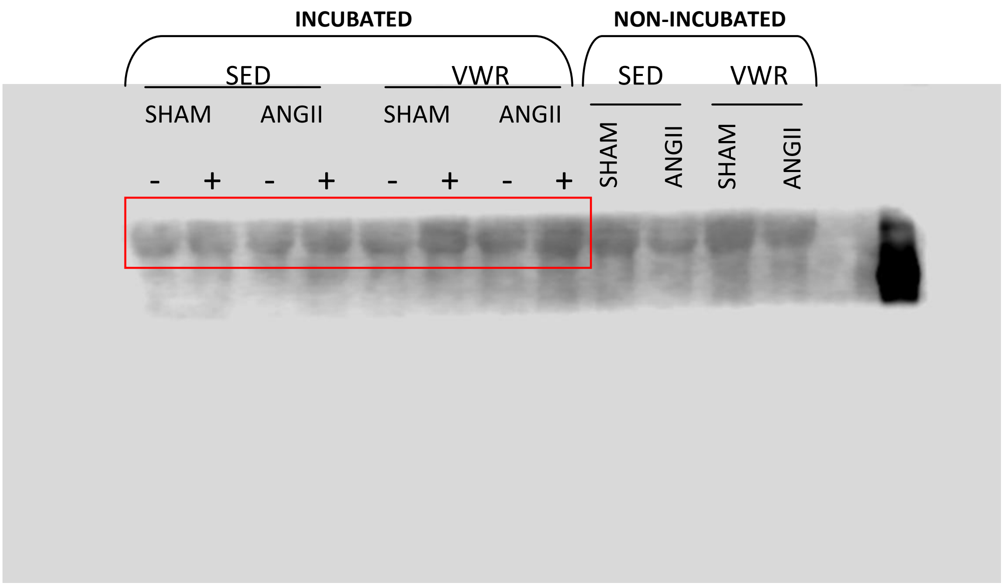

Figure 2.10 Expressions of AS160 in incubated soleus muscle

3. Original gels of expressions of 4-HNE protein in non-incubated soleus muscle represent in Figure 5A and MAPKs protein in non-incubated and incubated soleus muscle represent in Figure 5B and 5C respectively.

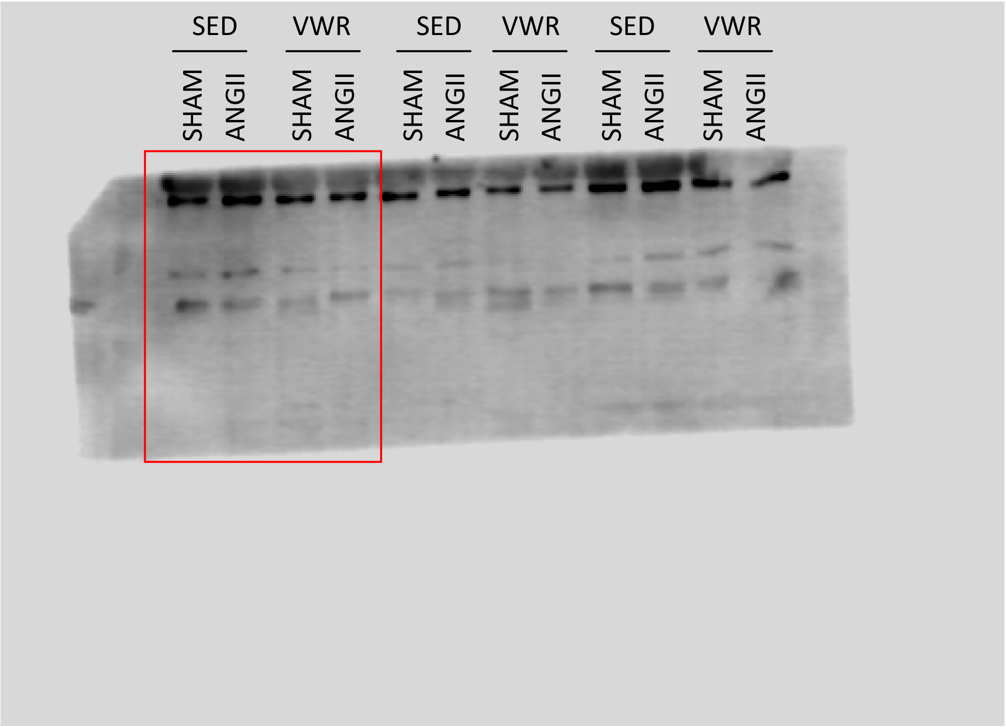

Figure 3.1 Expressions of 4-HNE protein in non-incubated soleus muscle

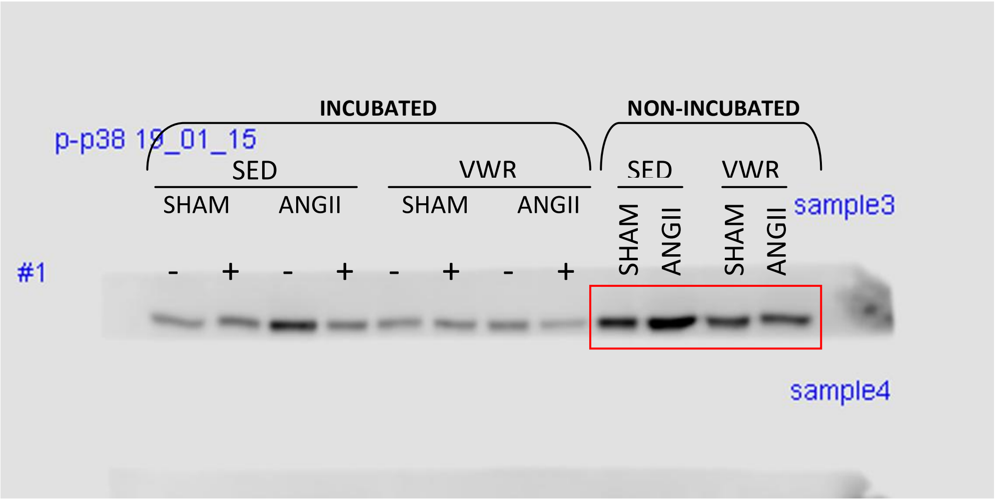

Figure 3.2 Expressions of phosphorylated p38 MAPK (Thr[180]/Tyr[182]) (p-p38 MAPK) in non-incubated soleus muscle

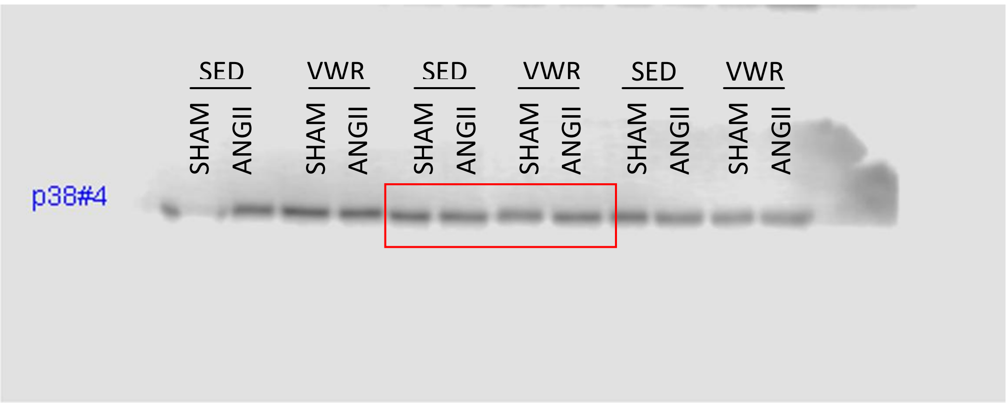

Figure 3.3 Expressions of p38 MAPK in non-incubated soleus muscle

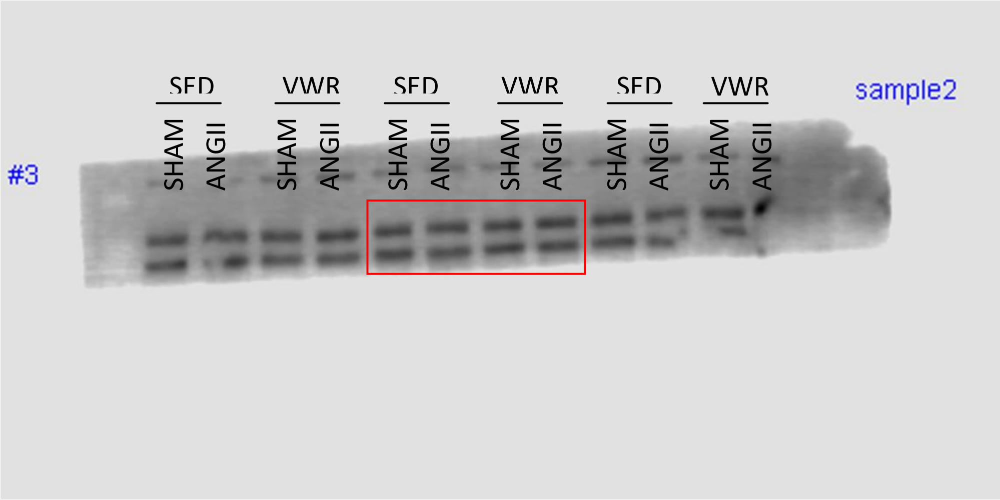

Figure 3.4 Expressions of phosphorylated SAPK/JNK (Thr^{183}/Tyr^{185}) (p-SAPK/JNK) in non-incubated soleus muscle

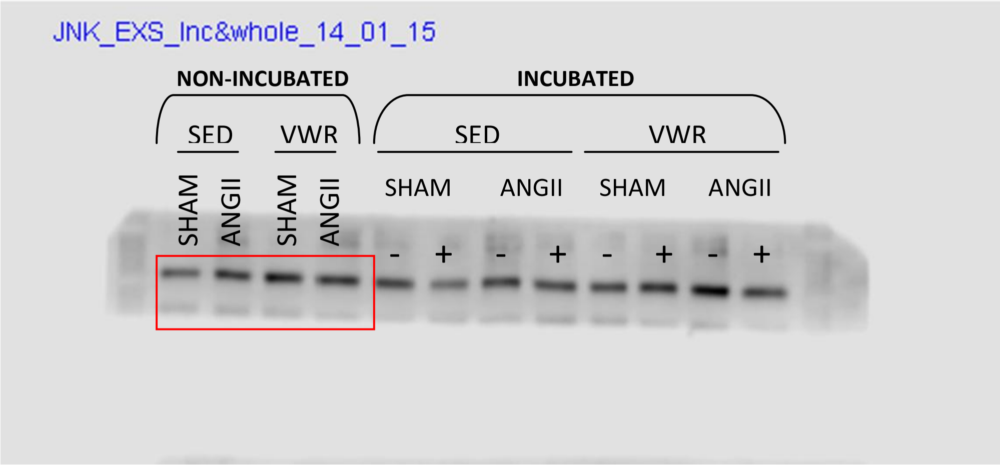

Figure 3.5 Expressions of SAPK/JNK in non-incubated soleus muscle

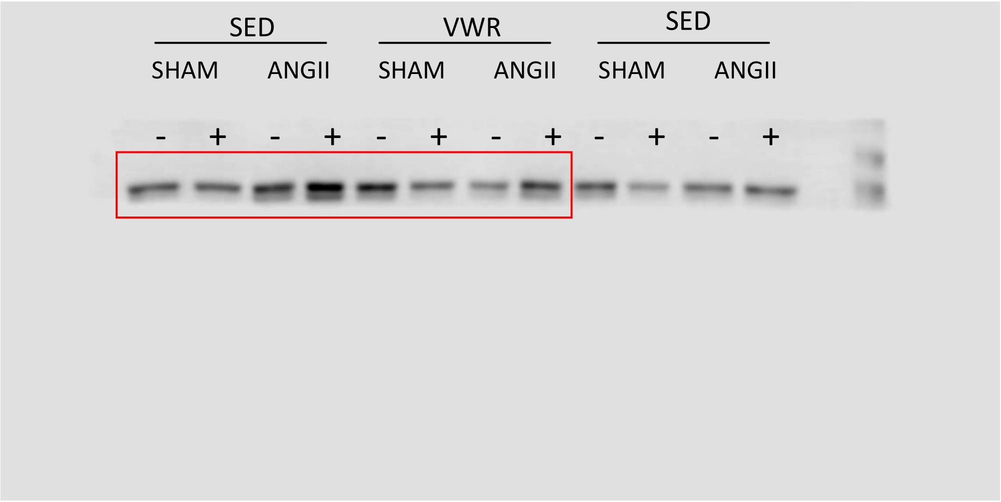

Figure 3.6 Expressions of phosphorylated p38 MAPK (Thr^{180}/Tyr^{182}) (p-p38 MAPK) in incubated soleus muscle

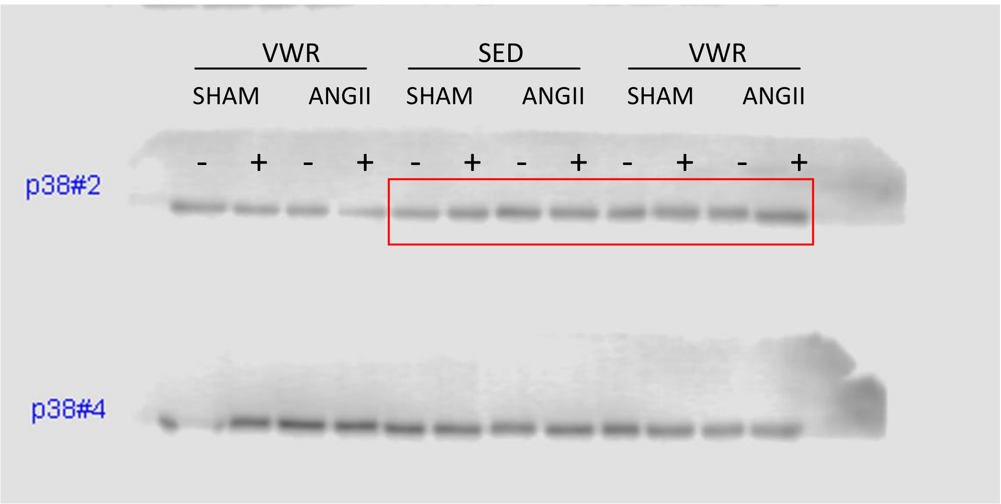

Figure 3.7 Expressions of p38 MAPK in incubated soleus muscle

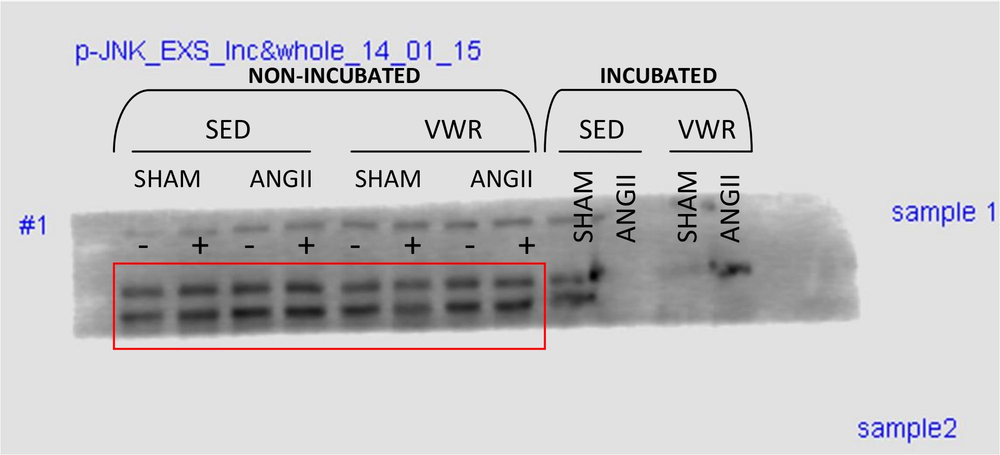

Figure 3.8 Expressions of phosphorylated SAPK/JNK (Thr[183]/Tyr[185]) (p-SAPK/JNK) in incubated soleus muscle

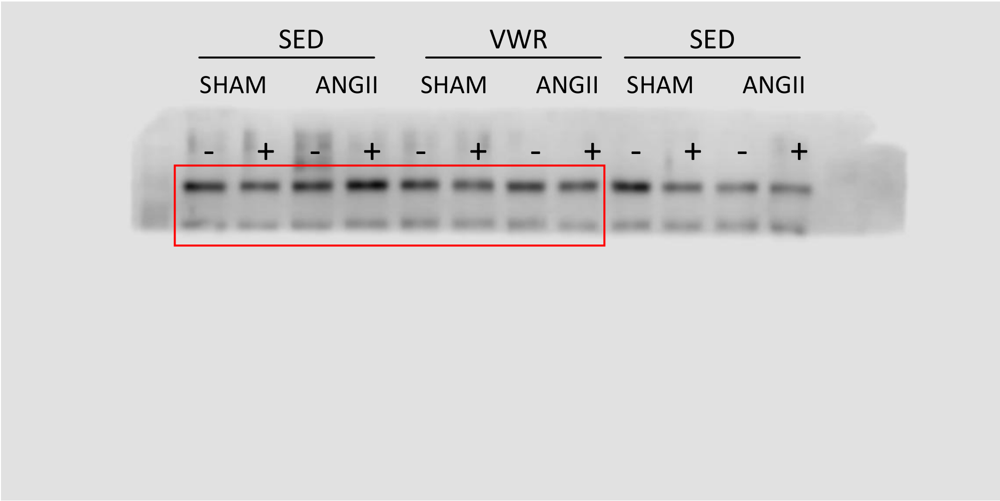

Figure 3.9 Expressions of SAPK/JNK in incubated soleus muscle

4. Original gels of expressions of AMPK and phosphorylated AMPK Thr[172] (p-AMPK Thr[172]) in Figure 6A, expressions of ACE1, AT1R and AT2R Figure 6B) and ACE2 and MAS receptor (MasR) in Figure 6C in non-incubated soleus muscle.

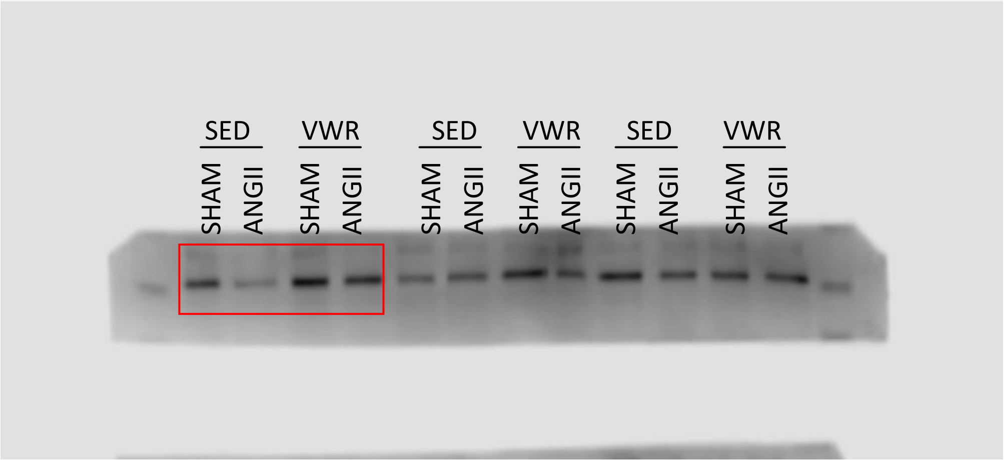

Figure 4.1 Expressions of phosphorylated AMPK Thr[172] (p-AMPK Thr[172]) in non-incubated soleus muscle

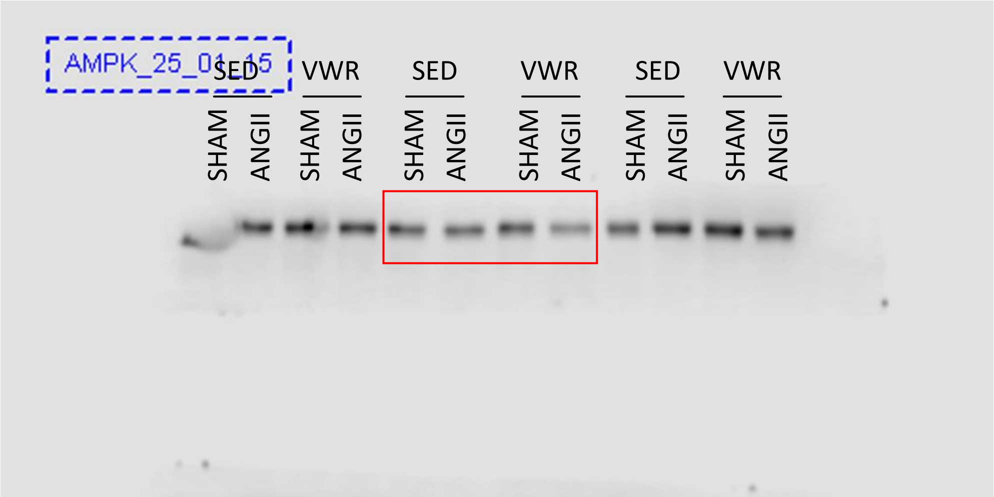

Figure 4.2 Expressions of AMPK in non-incubated soleus muscle

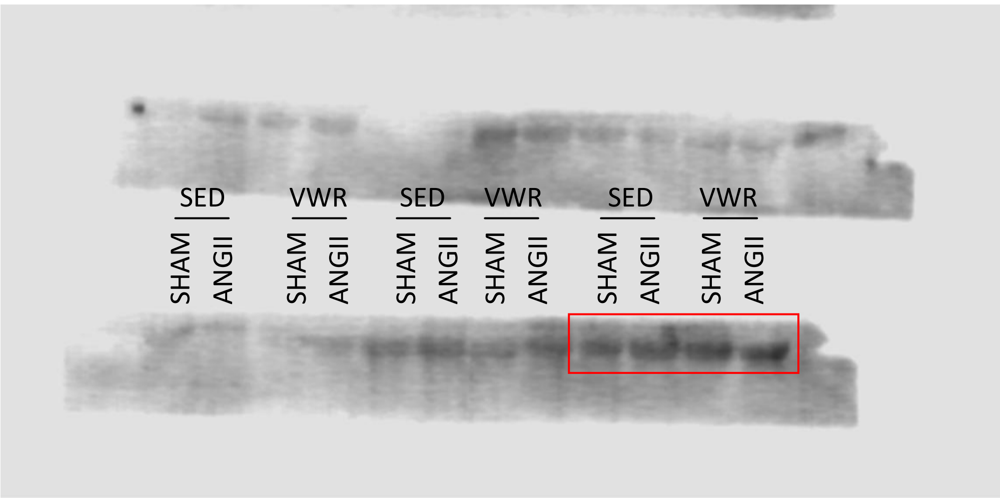

Figure 4.3 Expressions of ACE1 in non-incubated soleus muscle

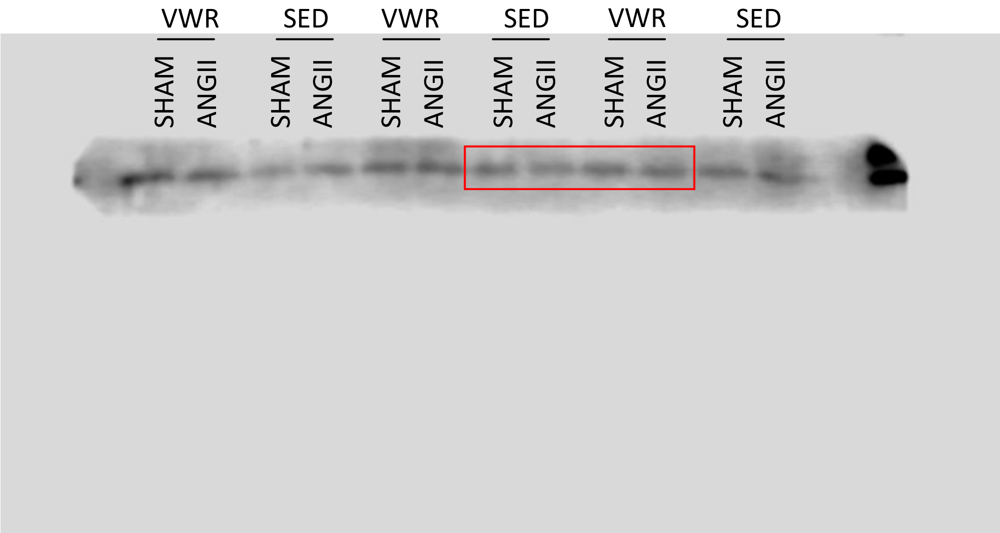

Figure 4.4 Expressions of AT1R in non-incubated soleus muscle

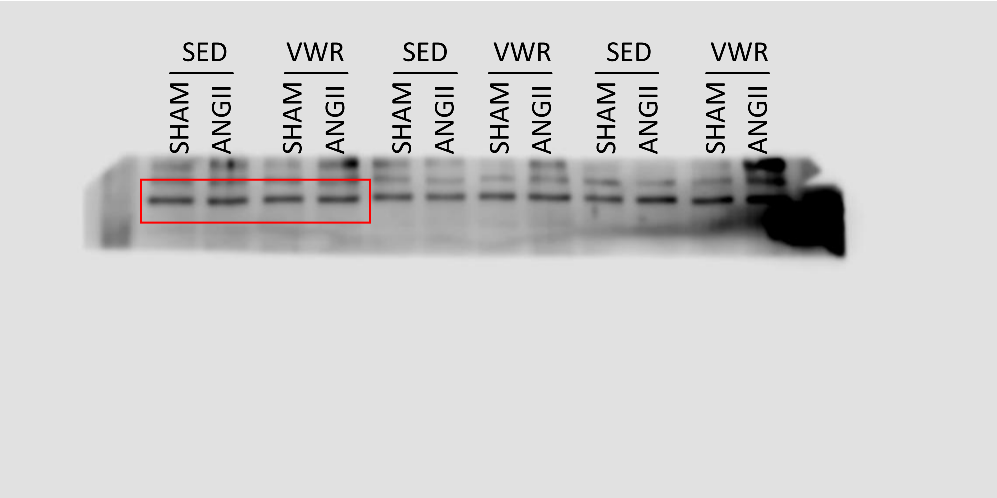

Figure 4.5 Expressions of AT2R in non-incubated soleus muscle

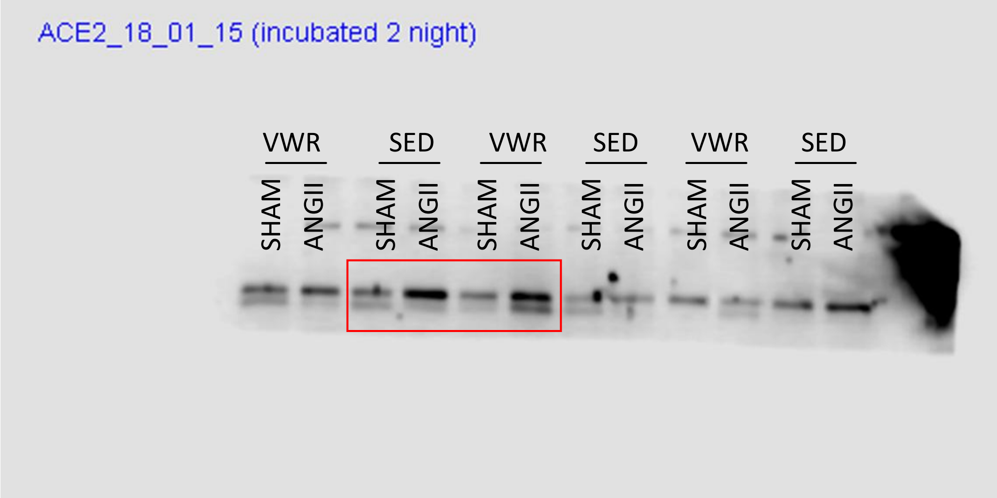

Figure 4.6 Expressions of ACE2 in non-incubated soleus muscle

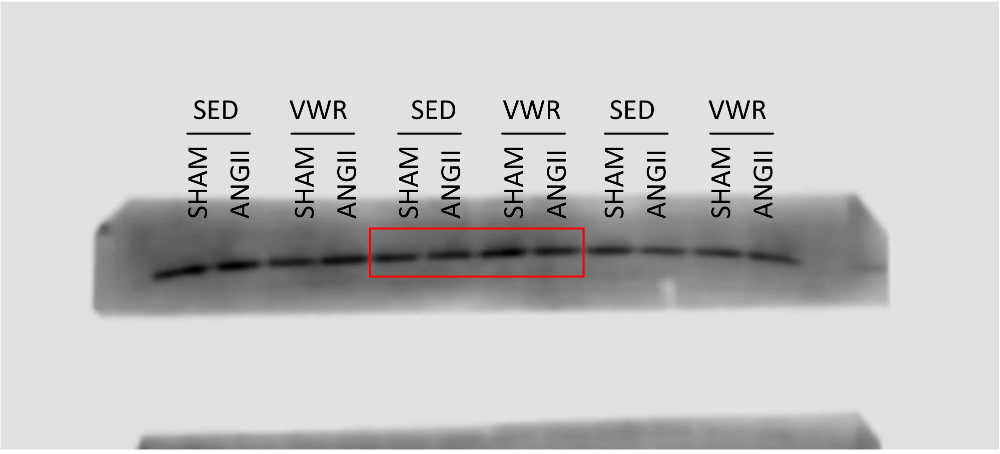

Figure 4.7 Expressions of MasR in non-incubated soleus muscle
